# Transcriptome-wide characterization and functional analysis of *Xyloglucan endo-transglycosylase/hydrolase* (*XTH*) gene family of *Salicornia europaea* L. under salinity and drought stress

**DOI:** 10.1186/s12870-021-03269-y

**Published:** 2021-10-25

**Authors:** Richard John Tiika, Jia Wei, Guangxin Cui, Yanjun Ma, Hongshan Yang, Huirong Duan

**Affiliations:** 1 Lanzhou Institute of Husbandry and Pharmaceutical Science, Chinese Academy of Agricultural Sciences, Lanzhou, China; 2 College of Forestry, Gansu Agricultural University, Lanzhou, China

**Keywords:** *Salicornia europaea*, PacBio Iso-Seq, *XTH* gene family, Salinity, Drought

## Abstract

**Background:**

*Salicornia europaea* is a halophyte that has a very pronounced salt tolerance. As a cell wall manipulating enzyme, xyloglucan endotransglycosylase/hydrolase (XTH) plays an important role in plant resistance to abiotic stress. However, no systematic study of the *XTH* gene family in *S. europaea* is well known. PacBio Iso-Seq transcriptome sequence data were used for bioinformatics and gene expression analysis using real-time quantitative polymerase chain reaction (RT-qPCR).

**Results:**

Transcriptome sequencing (PacBio Iso-Seq system) generated 16,465,671 sub-reads and after quality control of Iso-Seq, 29,520 isoforms were obtained with an average length of 2112 bp. A total of 24,869 unigenes, with 98% of which were obtained using coding sequences (CDSs), and 6398 possible transcription factors (TFs) were identified. Thirty-five (35) non-redundant potential SeXTH proteins were identified in *S. europaea* and categorized into group I/II and group III based on their genetic relatedness. Prediction of the conserved motif revealed that the DE(I/L/F/V)DF(I)EFLG domain was conserved in the *S. europaea* proteins and a potential N-linked glycosylation domain N(T)V(R/L/T/I)T(S/K/R/F/P)G was also located near the catalytic residues. All *SeXTH* genes exhibited discrete expression patterns in different tissues, at different times, and under different stresses. For example, 27 and 15 *SeXTH* genes were positively expressed under salt stress in shoots and roots at 200 mM NaCl in 24 h, and 34 *SeXTH* genes were also positively regulated under 48 h of drought stress in shoots and roots. This indicates their function in adaptation to salt and drought stress.

**Conclusion:**

The present study discovered *SeXTH* gene family traits that are potential stress resistance regulators in *S. europaea*, and this provides a basis for future functional diversity research.

**Supplementary Information:**

The online version contains supplementary material available at 10.1186/s12870-021-03269-y.

## Background

Plants regularly face numerous environmental discrepancies that include abiotic and biotic stresses, and abiotic stresses tend to occur frequently in their life cycle. Salinity stress and drought including low temperature are serious problems for plant growth and productivity as their full genetic potential is prevented by these harsh environmental factors [1, 2]. Salinity is one of the most detrimental factors limiting plant growth and leading to nutritional limitations by reducing the absorption of calcium, nitrate, potassium and phosphorus and causing osmotic stress [3, 4]. Drought is a gradual soil water depletion and potentially a major source of stress that can suppress crop growth and it is responsible for significant yield losses in agricultural crops [1, 5]. In response to abiotic stresses such as dehydration and excessive osmotic stress, plants resort to numerous adaptive strategies. These adaptive mechanisms involve changes in physiological and biochemical processes [6]. Consequently, most research on water stress signaling has focused on salt stress because plant responses to salt and drought stress are closely linked and signaling pathways overlap [2]. Many genes induced by salt and drought stress are part of a larger sequence of molecular networks and likely play important roles in environmental stress responses. These genes are involved in a variety of cellular and physiological function, including signal recognition and transmission, photosynthesis and energy metabolism, membrane transport, and protein production [7–11]. Numerous essential genes involved in multiple molecular pathways, such as *AtSOS1*, *AtNHX1*, *AtHKT1;1*, and *PutHKT2;1*, appear to be involved in plant responses to environmental stress [12–15]. Nevertheless, very few genes for salt and drought tolerance have been found so far, and most research on plant stress tolerance has focused on model plants such as *Arabidopsis thaliana*, *Oryza sativa*, as well as *Nicotiana tabacum* [16, 17].

*Salicornia europaea*, a succulent halophyte belonging to the family of *Amaranthaceae*, has many common names: Glasswort, Marsh samphire, and Saltwort. From available literature, this plant is one of the most salt-tolerant plants in the world [18]. As a result of evolutionary adaptation to harsh environments in unfavorable seasons, it exhibits very distinct salt and water tolerance abilities [19]. The optimal growth salinity of *S. europaea* is 200 mM and can tolerate soil concentrations greater than 1000 mM [3, 4, 20, 21]. *S. europaea* is ideal for cultivation to desalinate saline soils in extremely saline environments [22]. This halophyte can also be used for the development of biofuel precursors [23, 24] and as a source of secondary metabolites [25]. It is not only an important economic plant but also a suitable model plant for the identification of genes involved in abiotic stress tolerance mechanisms. Recently, salt tolerance processes and the accumulation of high salt concentrations in shoots of *S. europaea* have been studied by modifying proteomic signaling pathways under salt stress and identifying some genes related to salt tolerance [3, 17]. Although to our knowledge, *S. europaea* is one of the most salt-tolerant halophytes, only a limited number of genes of this plant have been identified and characterized under environmental stress [26, 27]. Therefore, it was very important to perform a comprehensive transcriptomic analysis of the *XTH* gene family under drought and salt stress. Xyloglucan endotransglycosylase/hydrolase (XTH) is one of the most important enzymes involved in biological processes [28, 29]. The XTH is a cell wall manipulating enzyme encoded in several genes, belonging to a group of glycoside hydrolase 16 (GH16) family [30]. It is involved in various physiological and biological processes, mainly plant growth and resistance to stress [31]. XTH typically perform two distinct catalytic activities: xyloglucan endo-hydrolase (XEH) and xyloglucan endo-transglycosylase (XET), with different influences on xyloglucan: XET action results in non-hydrolytic cleavage and shortening of the xyloglucan chain, whereas XET causes irreversible chain shortening [29, 31]. The highly conserved GH16 domain of XET (GH16-XET) shares XTH proteins with a specific ExDxE motif, which is thought to be the catalytic site for both XET and XEH activities. Thus, it contains the major C-terminal domain of XET (C-XET), which distinguishes the XTH members from all other GH16 subfamilies [32, 33]. The *XTH* subfamily is divided into three main groups (groups I/II and III), with XET activity being particularly pronounced in groups I and II, and group III being divided into two subgroups: IIIA and IIIB [34]. While group IIIB exhibited high XET activity, group IIIA showed prominent XEH activity [35, 36]. The expansion of the XTH family based on sequence similarity extends an ancestral group to different organisms [37].

Using publicly available datasets, a growing number of *XTH* genes have been identified [34]. For instance, *A. thaliana* (33), *O. sativa* (29), *Solanum lycopersicum* (25), *N. tabacum* (56), *Glycine max* (61), and *Hordeum vulgare* (24) have been identified as potential *XTH* members [17, 35, 36, 38–40]. The *XTH* gene has recently been shown to be of particular interest as unique gene members have been identified that influence plant responses to abiotic stresses [38, 40–43]. For example, *CaXTH3* from *Capsicum annuum* was found to be highly expressed in transgenic *A. thaliana* lines and to confer increased resistance to salt and drought stress [40, 43]. *MtXTH3* was strongly up-regulated in *Medicago truncatula* at higher NaCl concentration [36]. In *A. thaliana*, *AtXTH14, − 15,* and *− 31* showed significantly reduced expression when exposed to aluminum stress, especially *AtXTH31* [43].

RNA sequencing (RNA-Seq) is a promising application of next-generation sequencing that has been successfully used for whole transcriptome analysis in most non-model plants that lack a reference genome [44–46]. Using this method, researchers can identify transcriptome structures, novel transcripts, genes that are differentially expressed, alternative splicing and genetic variants [47]. Short-read sequencing using the Illumina platform is a powerful method for quantifying gene expression. However, the limitations of short-read sequencing lead to a number of computational challenges and hamper transcript reconstruction and splicing event detection. In recent years, an increasing number of full-length Pac-Bio transcriptomes have been generated. The PacBio Iso-Seq (isoform sequencing) platform yields long reads often up to 10 kb, which enables accurate reconstruction of full-length splice variants [45, 48–50]. Due to the limitation of genomic data in *S. europaea*, only transcriptome data are publicly available, but these are Illumina sequencing transcriptome data [16, 24, 51], thus the PacBio Iso-Seq platform was used in this study.

Here, we used PacBio Iso-Seq to identify *XTH* genes of *S. europaea* associated with abiotic stress and performed some bioinformatics studies. To explore the possible involvement of *XTH* genes responding to NaCl and drought stress, the *S. europaea XTH* genes were exposed to salinity and drought stress. Expression of all identified *SeXTH* genes was validated by RT-qPCR. The dataset provides a comprehensive list of *SeXTH* genes and an overview of their dynamic expression patterns and their potential role in controlling plant resistance to abiotic stress. This work provides important insights into the functional role of *XTH* genes in *S. europaea* under stress conditions.

## Methods

### Plant materials

Wild seeds of *S. europaea* were collected from swampy areas of Liangcao Village, Jingtai County, Baiyin City, Gansu Province in China (37°21′2′′N, 104°5′28′′W). It is a widely distributed wild plant in Jingtai, thus collecting for scientific research in Gansu Province is not restricted. The voucher specimen was formally identified by a plant taxonomist, Fanglan He of Gansu Desert Control Research Institute, and kept in Lanzhou Institute of Husbandry and Pharmaceutical Science Herbarium (CYSLS-HrDuan20171130). The plant material collections and experimental research complied with local legislation, national and international guidelines.

Seeds were surface sterilized, washed, and grown for 3 d in Petri-dish under darkness. The plantlets were transferred to containers of sterilized sand and soaked in ½ Hoagland nutrient solution after germination. The plantlets were grown in a growth chamber at 25/22 °C with 65% relative humidity (day/night) and 16/8 h of light and darkness for 4 weeks. The pH of ½ Hoagland nutrient solution was standardized at 5.5 and was renewed every 3 days.

The four-week-old seedlings were subjected to NaCl and drought stress. The seedlings were exposed to NaCl (0, 50 and 200 mM) for periods of 0, 6, 24, and 48 h, respectively. To affect the water activity for drought stress, the seedlings were exposed to − 0.5 MPa D-sorbitol for 0, 6, 24, and 48 h, and the concentration of D-sorbitol was determined according to Lü et al. [50]. The shoots and roots were collected separately and quickly frozen in liquid nitrogen and stored at − 80 °C for further analysis.

The tissues of the shoots and roots of different NaCl concentrations (0, 50, 100, 200, 300 mM) treated in the period of 0, 6, and 24 h were collected and mixed together, and the weights were approximately equal. The mixed sample of the different tissues was then prepared for PacBio Iso-seq sequencing. The samples were immediately frozen in liquid nitrogen and stored at − 80 °C until use.

### RNA quantification and quality assessment for sequencing

A mirVana miRNA Isolation Kit (Thermo Fisher Scientific, Waltham, MA, USA) was used to extract total RNA. RNA degradation and contamination were assessed on one-percent agarose gels. The NanoDrop 2000 instrument (Thermo Fisher Scientific, Waltham, MA, USA) was used to assess RNA quantity and consistency, and an Agilent 2100 Bioanalyzer (Agilent Technologies, Santa Clara, CA, USA) was used to test RNA integrity.

### PacBio Iso-Seq library preparation and sequencing

The SMRTbell™ Template Prep Kit 1.0-SPv3 (Pacific Biosciences, Menlo Park, CA, USA) was used to run the sequencing library on 1 μg total RNA from the mixed shoot and root sample. The Qubit 2.0 fluorometer (Life Technologies, Carlsbad, CA, USA) was used to check the final library amount and concentration. An Agilent 2100 Bioanalyzer was used to assess library size and purity (Agilent Technologies, Santa Clara, CA, USA). Magbead-loaded SMRTbell template was run on a PacBio Sequel instrument at Shanghai Oe Biotech Co., Ltd. according to Sequel Binding Kit 2.0 instructions (Pacific Bioscience, USA) for primer annealing and polymerase binding (Shanghai, China).

### PacBio data analysis

After Iso-seq quality control (https://github.com/PacificBiosciences/IsoSeq SA3nUP/wiki#datapub), including circular consensus sequence (CCS) generation, classification, and cluster analysis, high-quality consensus isoforms were identified from the original sub-breads. Error correction of the combined high and low quality isoforms was performed using RNA-Seq data and LoRDEC software. Using the program TOFU (http://github.com/PacificBiosciences/cDNA primer/), high-quality transcripts were generated with an identification value of 0.85. BLASTX (E-value ≤1^e-5^) was used to search the non-redundant (NR), SWISS-PROT and Kyoto encyclopedia of genes and genomes (KEGG) [52] protein databases for all known non-redundant transcripts, and putative coding sequences (CDSs) were validated from the highest-ranked proteins. Using BLAST, non-redundant transcripts were aligned with the PlantTFDB (http://planttfdb.cbi.pku.edu.cn/index.php) and AnimalTFDB (http://bioinfo.life.hust.edu.cn/AnimalTFDB/) databases to obtain information on transcription factors (TFs).

### Identification of the XTH proteins of *S. europaea*

To identify the XTH proteins, this study used the current transcriptome sequences from *S. europaea*, using publicly available datasets from *A. thaliana, M. truncatula* and *N. tabacum* [17, 36, 53] as references. First, 24,869 unigenes from the transcriptome data were searched for all possible *S. europaea* XTH proteins and the possible XTH protein sequences were additionally identified using Blastp search. The online software SMART (http://smart.embl-heidelberg.de/) [54] was used to determine conserved domains of putative XTH proteins. HMMER version 3.3.2 was downloaded [55] and used to further validate the predicted protein data with the inclusion of *A. thaliana, M. truncatula* and *N. tabacum* as a queries; with E value set to 1e-20. The proteins with XTH domain were conserved for further study.

### Phylogenetic analysis, multiple sequence alignment and construction of conserved motifs

A phylogenetic tree of SeXTH proteins was constructed using MEGA 5.0 with 1000 bootstrap replicates from Maximum Likelihood [56], and AtXTHs from *A. thaliana* (40), NtXTHs from *N. tabacum* (50), and MtXTHs from *M. truncatula* (35) were used as queries (Supplementary file 1). The *A. thaliana* and *M. truncatula* protein sequences were obtained from the Arabidopsis Information Resource (TAIR) database (http://www.arabidopsis.org/) and the NCBI database (https://www.ncbi.nlm.nih.gov/), respectively, and the *N. tabacum* protein sequence was downloaded from Wang et al. [17]. ClustalW was used with default settings to perform multiple sequence alignment of candidate SeXTH proteins at the amino acid level [57]. Furthermore, the conserved motifs in SeXTH proteins were characterized using Multiple Expectation Maximization for Motif Elicitation (MEME) (http://meme-suite.org/doc/cite.html), examing a motif length of 6–50 sequences and a total of 10 motifs [58].

### RNA isolation, cDNA transcription, and RT-qPCR analysis for *SeXTH* genes

Total RNA was extracted from approximately 100 mg of the frozen shoot and root tissue using the TransZol Up Plus RNA Kit (Lot#M31018) according to the manufacturer’s instructions. RNA quantity and quality were examined using a TGen spectrophotometer (TianGen) based on the A260 nm/A280 nm and A260 nm/A230 nm ratios. The *Evo* M-MLV RT Kit (AG11705, Accurate Biotechnology) was used to reverse transcribe total RNA into cDNA and remove genomic DNA mixed in the cDNA, according to the manufacturer’s protocol.

RT-qPCR analysis was performed according to MIQE guidelines [59]. Primers were designed based on the mRNA sequences using Primer 5 software (Supplementary file 2) and synthesized by TsingKe Biological Technology Co., Ltd. (Xi’an, China). The *Ubiquitin-conjugating* (*UBC*) gene, which is an abiotic stress and tissue universal reference gene in *S. europaea*, was used as a housekeeping/internal control gene [60] and primer pair specificity was determined using RT-qPCR single peak melting curve analysis. Three independent biological replicates were performed and triplicate technical quantitative assays were performed with 0.5 μl of each cDNA dilution using the Heiff® qPCR SYBR® Green Master Mix kit (Yeasen Biotech Co., Ltd) according to the manufacturer’s protocol (Supplementary file 3). The RT-qPCR analysis was performed using the QuantStudio™ 5 Real-Time PCR instrument (ABI). The relative expression of *SeXTH* genes was calculated using the 2^−ΔΔCt^ method [61].

### Data analysis

Gene expression values are presented as means ± SD (*n* = 3), and the data were analyzed using SPSS statistical software (Ver. 22.0, SPSS Inc., Chicago, IL, USA) using the one-way ANOVA. At a significance level of *p* < 0.05, Duncan’s multiple range tests were used to detect differences between means and GraphPad Prism 8 was used to plot all graphs.

## Results

### Sequencing and analysis of the transcriptome of *S. europaea* using the PacBio Iso-Seq platform

A higher quality transcriptome assembly was obtained from the mixed samples of the different organs of *S. europaea*, which was synthesized using the PacBio Iso-Seq system and yielded 16,465,671 subreads. After Iso-Seq quality control, 29,520 isoforms were generated with an average length of 2112 bp. A total of 24,869 unigenes were generated with an average length of 2101 bp. Using the annotation information, 6398 possible TFs were predicted. The unigenes were used to predict the coding region sequences after BLASTX search. The results yielded 24,414 unigenes predicted by CDS (98% of the total unigenes) and a total of 182 interspersed repeats with a total length of 10,989 bp. The raw sequence data were submitted to the NCBI Sequence Read Achieve database with accession number: PRJNA725943 (https://submit.ncbi.nlm.nih.gov/subs/sra).

### Identification of *S. europaea* XTH proteins

Generally, members of XTH family are conserved in various species. Therefore, 24,869 unigenes were searched from the transcriptome data of *S. europaea* for all possible SeXTH proteins, and 35 best non-redundant potential candidates were obtained and confirmed by BLAST on NCBI database (Supplementary file 4) and the HMMER version 3.3.2 software (Supplementary file 5). They were renamed from SeXTH1 to SeXTH35, respectively. It was discovered that SeXTH4/5, SeXTH7/8, SeXTH10/11, SeXTH26/27 and SeXTH33/34 were different transcripts of the same genes. The predicted length of the 35 SeXTH proteins revealed that SeXTH16 and − 17 had the longest protein sequence with 384 amino acids, and the smallest was SeXTH9 with 127 amino acids (Table 1).

**Table 1:** The statistics information of 35 SeXTH proteins from *S. europaea*

Name	Transcript ID	Length (Aa)	Catalytic Site
SeXTH1	i0_HQ_samplee11669_c1714_f3p2_980	278	DEIDFEFLG
SeXTH2	i1_HQ_samplee11669_c284462_f3p3_1081	262	DEIDFEFLG
SeXTH3	i1_HQ_samplee11669_c455308_f7p4_1100	284	DEIDFEFLG
SeXTH4	i0_HQ_samplee11669_c526_f5p0_971	269	DEIDFEFLG
SeXTH5	i1_HQ_samplee11669_c498512_f29p7_1226	281	DEIDFEFLG
SeXTH6	i1_HQ_samplee11669_c456195_f2p5_1122	242	DEIDFEFLG
SeXTH7	i1_HQ_samplee11669_c413025_f29p7_1199	283	DEIDFEFLG
SeXTH8	i1_HQ_samplee11669_c500149_f6p6_1113	283	DEIDFEFLG
SeXTH9	i2_HQ_samplee11669_c21901_f2p2_2292	127	–
SeXTH10	i1_HQ_samplee11669_c9245_f4p1_1355	285	DELDFEFLG
SeXTH11	i1_HQ_samplee11669_c175741_f4p1_1292	285	DELDFEFLG
SeXTH12	i1_HQ_samplee11669_c148489_f3p1_1490	338	DELDFEFLG
SeXTH13	i1_HQ_samplee11669_c33309_f3p1_1216	343	DELDFEFLG
SeXTH14	i1_HQ_samplee11669_c75909_f61p8_1200	189	DEIDFEFLG
SeXTH15	i1_HQ_samplee11669_c175468_f17p8_1201	283	DEIDFEFLG
SeXTH16	i1_HQ_samplee11669_c323900_f11p2_1862	384	DEIDFEFLG
SeXTH17	i1_HQ_samplee11669_c324363_f6p2_1657	384	DEIDFEFLG
SeXTH18	i1_HQ_samplee11669_c456730_f3p0_126	292	NEFDFEFLG
SeXTH19	i1_HQ_samplee11669_c413221_f5p0_1297	290	NEFDFEFLG
SeXTH20	i1_HQ_samplee11669_c456125_f4p5_1076	269	DEIDFEFLG
SeXTH21	i3_HQ_samplee11669_c15535_f2p3_3018	269	DEIDFEFLG
SeXTH22	i1_HQ_samplee11669_c6078_f5p0_1381	342	DELDIEFLG
SeXTH23	i1_HQ_samplee11669_c500266_f6p1_1044	282	DEIDFEFLG
SeXTH24	i1_HQ_samplee11669_c501908_f3p1_1100	284	DEIDFEFLG
SeXTH25	i1_HQ_samplee11669_c119883_f27p1_1259	296	DEIDMEFLG
SeXTH26	i0_HQ_samplee11669_c858_f7p2_997	159	DEIDFEFLG
SeXTH27	i1_HQ_samplee11669_c243646_f13p5_1193	159	DEIDFEFLG
SeXTH28	i1_HQ_samplee11669_c45533_f5p5_1441	236	DEVDFEFLG
SeXTH29	i1_HQ_samplee11669_c498_f39p5_1467	289	DEVDFEFLG
SeXTH30	i1_HQ_samplee11669_c322708_f7p5_1222	216	DEIDFEFLG
SeXTH31	i1_HQ_samplee11669_c499858_f10p6_1142	247	DEIDFEFLG
SeXTH32	i1_HQ_samplee11669_c9096_f2p5_1303	176	–
SeXTH33	i1_HQ_samplee11669_c134347_f3p1_1137	144	DEIDFEFLG
SeXTH34	i1_HQ_samplee11669_c581117_f2p1_1026	144	DEIDFEFLG
SeXTH35	i1_HQ_samplee11669_c414106_f24p2_1215	149	DEIDFEFLG

### Phylogenetic analysis of SeXTH proteins

**Fig. 1:**
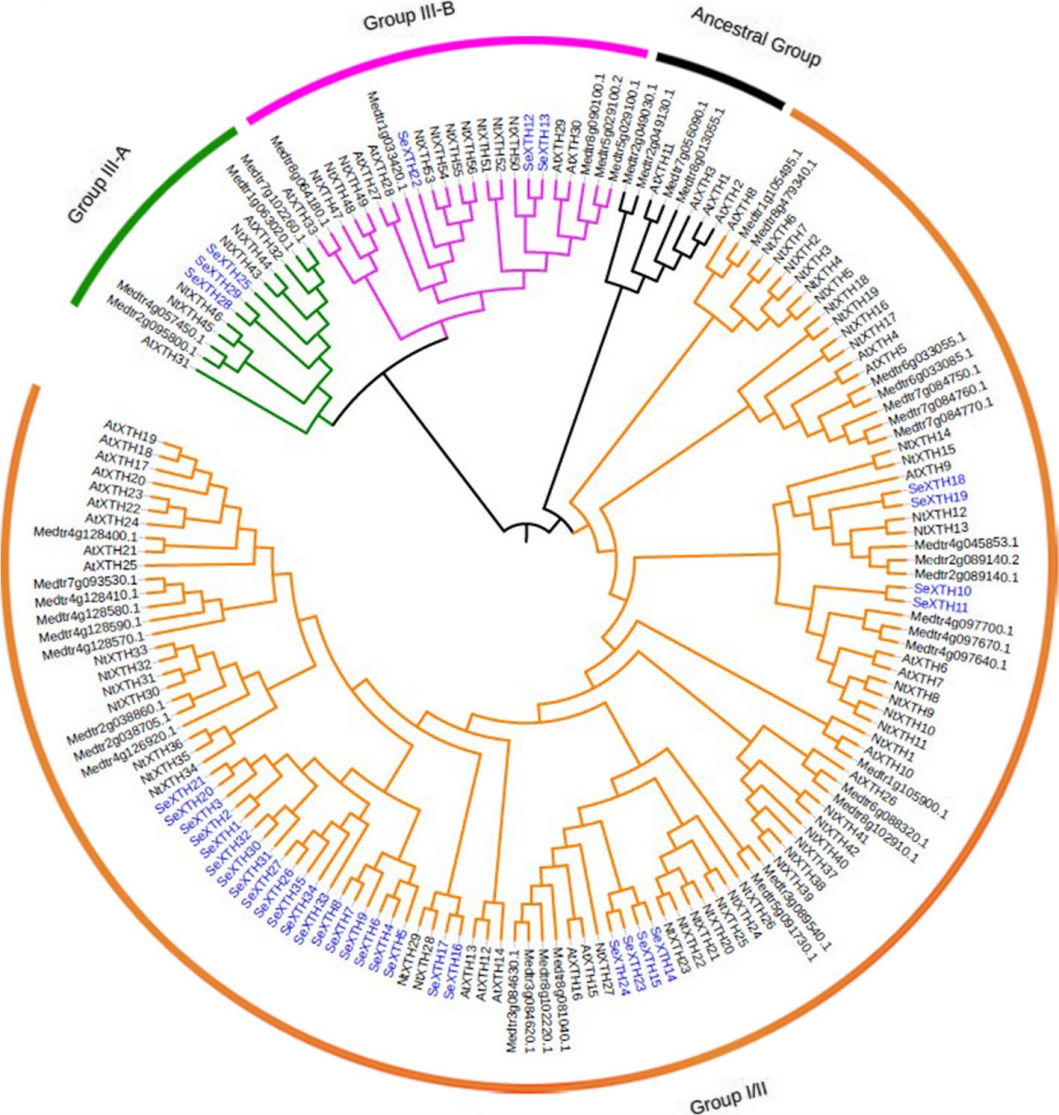
Phylogenetic tree of the proteins of *S. europaea, A. thaliana*, *M. truncatula* and *N. tabacum*. The different colored branches and arcs show Group I/II, IIIA, IIIB and Ancestral Group, respectively, with SeXTH members highlighted in blue. SeXTHs represent XTH members from *S. europaea*, AtXTHs represent *A. thaliana* members and *M. truncatula* members represent Medtrxxxx

Phylogenetic analysis of plant XTH proteins is a reliable way to understand the functions of unknown XTH members based on their genetic history and sequence consistency. Therefore, all thirty-five (35) SeXTH proteins identified with ExDxE domains were used to construct the phylogenetic relationship tree. The SeXTH proteins were clustered into group I/II, IIIA and IIIB. The majority of the proteins clustered into group I/II, which included twenty-nine (29) members. Similarly, group III consisted of SeXTH12, − 13, − 22, − 25, − 28, and − 29 and was further subdivided into group IIIA and group IIIB as defined previously [62, 63]. In *S. europaea*, no XTH protein was detected in the ancestral group (Fig. 1).

### Multiple sequence alignment of SeXTH proteins

**Fig. 2:**
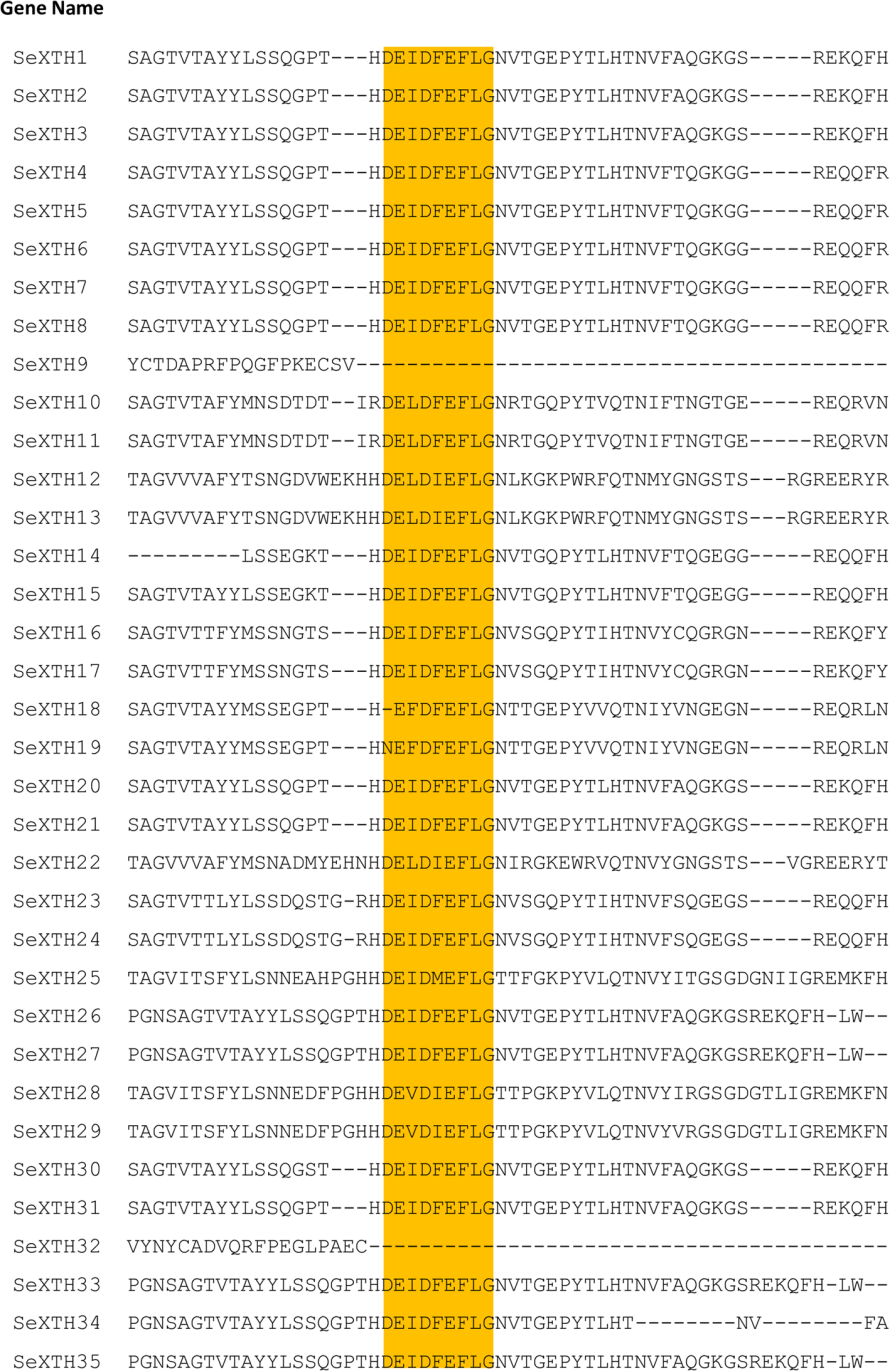
Multiple sequence alignment of the 35 SeXTH proteins. Missing amino acids are represented by dashes, while conserved XTH domains are highlighted in gold

The active site (ExDxE), which represents the catalytically active residues, was highly conserved in SeXTH proteins. In our study, the conserved domain DE(I/L/F/V)D(F/I/M)EFLG was identified in all SeXTH proteins except SeXTH9 and − 32, which were, however, confirmed to be members of the glycoside hydrolase 16 family by HMMER analysis. A potential N-linked glycosylation site sharing N(T)V(R/L/T/I)T(S/K/R/F/P)G is located near the catalytic residues in 33 SeXTH proteins (Table 1, Fig. 2).

### Analysis of conserved motifs in SeXTH proteins

**Fig. 3:**
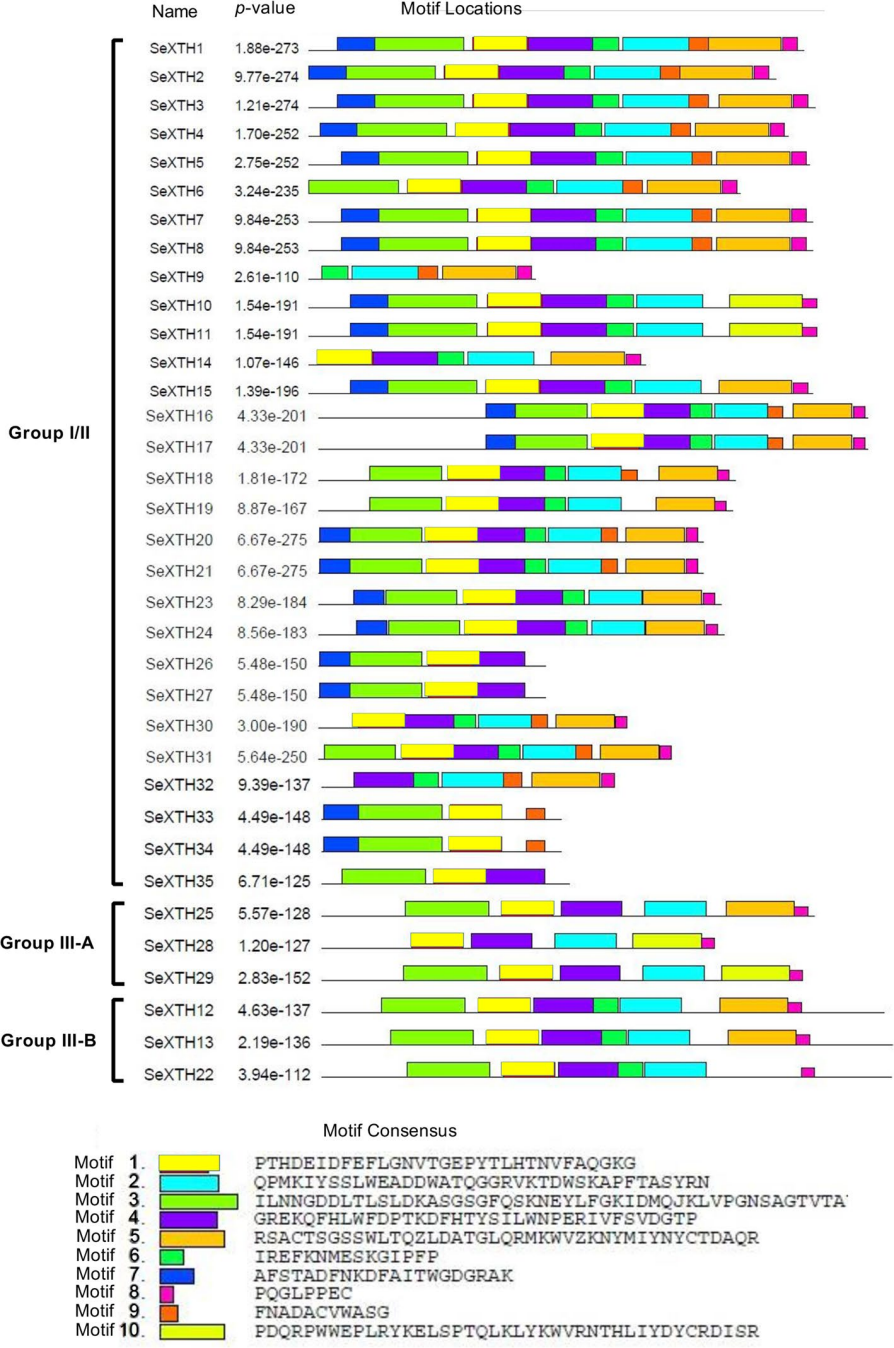
Conserved XTH protein motifs in *S. europaea*. The motif composition of the SeXTH protein was identified using MEME. The different colored boxes represent different motifs and their position in each SeXTH sequence. Each motif is indicated by a colored box in the legend at the bottom

We examined the diversity of motifs in the regions of SeXTH proteins using the MEME database (http://meme.nbcr.net/meme/). Ten (10) types of motifs from *S. europaea* were identified*.* Analysis revealed that motif 1 contained the conserved ExDxE active site. Importantly, motif 3 had the highest amino acid sequences, while motif 8 contained the fewest amino acid sequences. The majority of SeXTH proteins contained most of the motifs. For example, 11 SeXTH proteins contained 9 types of motifs and 8 SeXTH proteins contained 8 types of motifs. Five SeXTH proteins had 6 types of motifs, four SeXTH proteins contained 7 and 4 types of motifs, respectively, two SeXTH proteins had 5 types of motifs, and only 1 SeXTH had 3 types of motifs. Motifs 7 and − 9 were present only in group I/II. The different types of motifs exhibited by the SeXTH proteins were not defined (Fig. 3).

### Expression pattern of *SeXTH* genes in response to NaCl stress by RT-qPCR

**Fig. 4:**
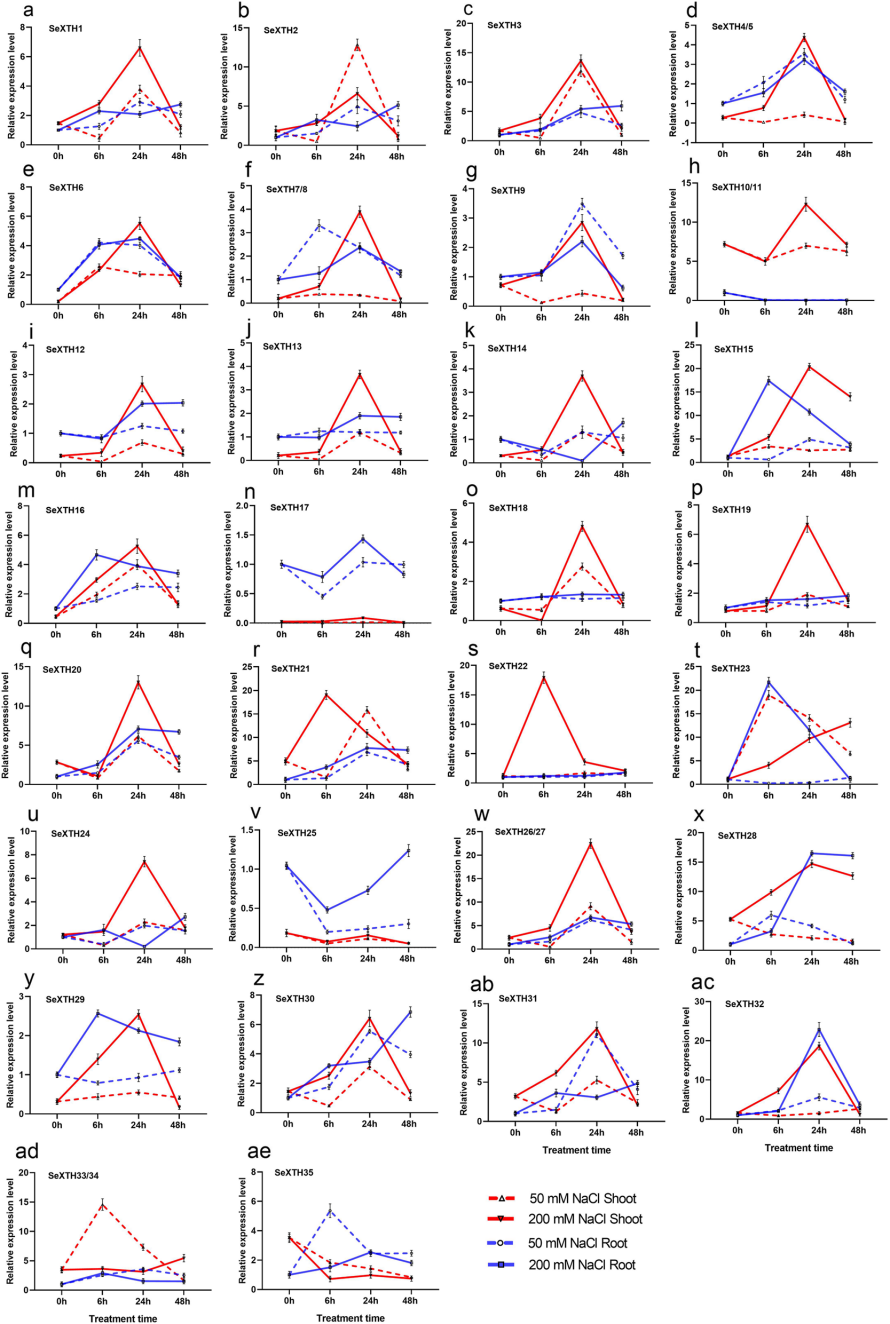
RT-qPCR analysis of the expression of 35 *SeXTH* genes in response to NaCl stress. The red and blue dashed lines represent 50 mM NaCl for shoot and root; the continued red and blue lines indicate 200 mM NaCl for shoot and root. The name of the gene is indicated at the top left top of each line graph

To investigate the relative expression pattern of *SeXTHs* in response to different abiotic stress conditions, the expression patterns of the 35 *SeXTHs* in response to salinity were examined and shown in Fig. 4. This analysis revealed that some members of the Se*XTH* genes have distinct tissue-specific expression profiles. For example, within group I/II, *SeXTH10/11* was mainly expressed in roots and *SeXTH17* in shoots. In addition, individual *SeXTH* genes responded differently to NaCl stress and time: 24 h recorded the highest number of members (15 *SeXTHs*) significantly expressed in all treatments and tissues, with an average fold-change of 5.7 compared to control. Under 0, 50, and 200 mM NaCl, 27 and 15 *SeXTHs* were expressed in shoots and roots respectively, after 24 h (Fig. 4).

### Expression pattern of *SeXTH* genes in response to drought stress by RT-qPCR

**Fig. 5:**
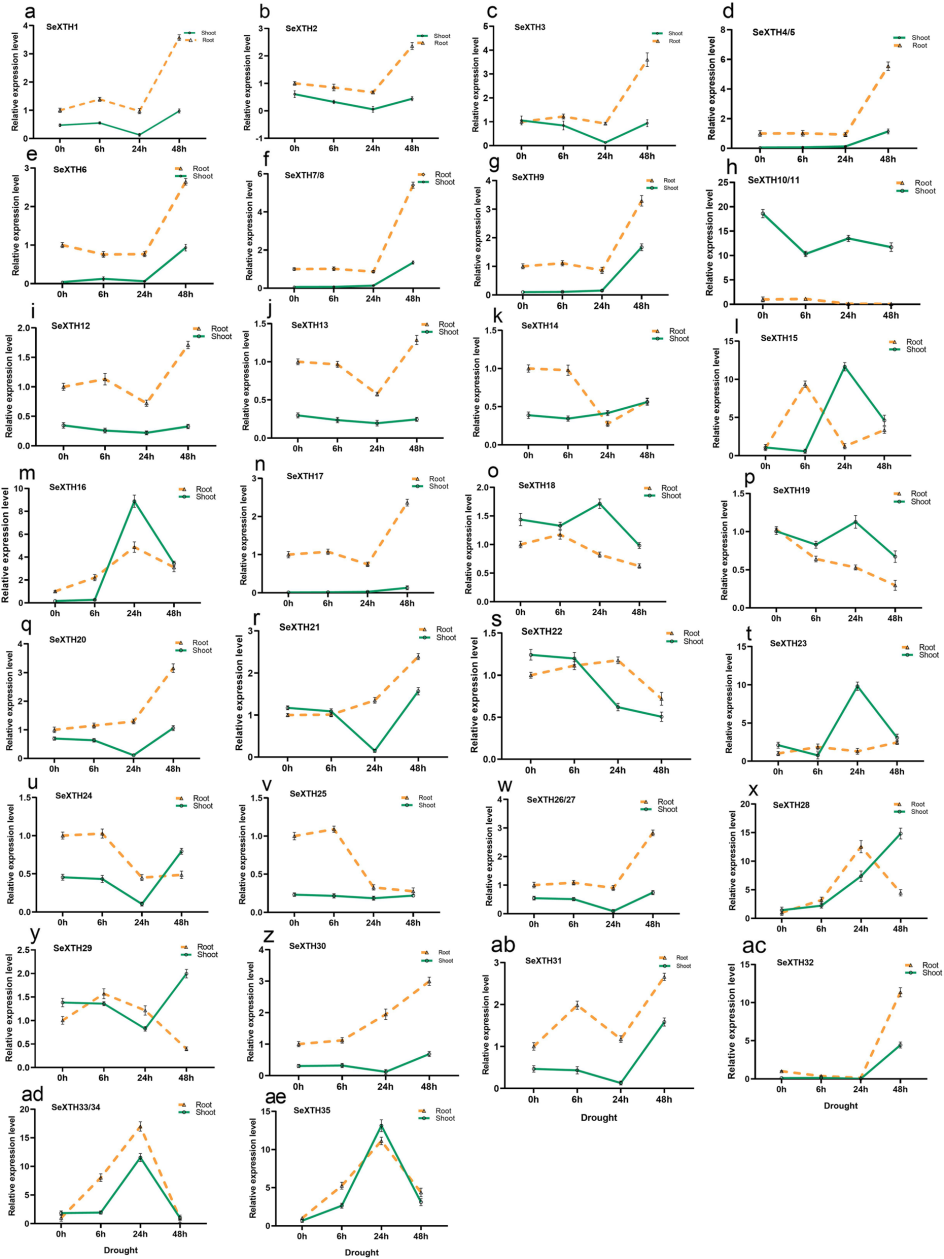
RT-qPCR analysis of the expression of 35 *SeXTH* genes in response to drought stress. The dashed lines represent roots and the continuous lines indicate shoots. The name of the gene is indicated at the top left of each line graph

Under drought, 21 *SeXTHs* showed statistically significant expression patterns in roots after 48 h of drought stress with 2.6, 1.4, 2.6, 4.5, 1.6, 4.4, 2.3, 0.7, 0.3, 2.4, 2.1, 1.4, 2.2, 1.4, 1.5, 1.8, 3.5, 2.0, 1.7, 10.4, 3.4 times higher compared to control. The expression levels of *SeXTH16, − 22, − 28, − 33/34* and − *35* were highly increased in roots after 24 h. In shoots, 13 *SeXTHs* were up-regulated and the expression levels of *SeXTH4/5, − 7/8, − 9, − 31* and *− 32* were down-regulated at the onset of drought stress and then increased after 48 h. *SeXTH16, − 23, − 28, − 33/34,* and *− 35* were also significantly increased after 24 h with fold-change of 7.9, 8.8, 1.2, 10.6, and 12.1, respectively compared to the control. The remaining genes showed different expression patterns in both roots and shoots depending on the duration of drought (Fig. 5).

## Discussion

Several non-model plants that thrive in harsh environments have evolved specialised mechanisms to survive that are of great value to scientific research. In species without a published genome or with an incomplete sequenced genome, such as *S. europaea*, the phenomenon of PacBio Iso-Seq transcriptome sequencing facilitates the study of transcriptomes and the expression profiling of relevant genes [24, 47]. In the present study, 24,869 high-quality unigenes were generated by PacBio sequencing and 24,414 unigenes were predicted using CDSs (98% of the total unigenes). Using the annotation information, 6398 possible TFs were predicted while another study on *S. europaea* discovered 171 and 143 transcription factors among DEGs in roots and shoots, respectively [24]. In walnut (*Juglans regia*), Sadat-Hasseini et al. [47] reported 3931 TFs when transcripts were searched using BLAST search. The unigene and annotation data in this study had higher quality. This suggests that the unigene data are of higher quality and suitable for gene identification, characterization and expression analysis. To the best of our knowledge, only three studies have investigated transcriptome profiling of *S. europaea*. These three previous studies had used the Illumina sequencing technique for transcriptome profiling, and although they generated the largest number of unigenes, this study used PacBio Iso-Seq to capture the most coding sequence unigenes and also the longest length unigenes [16, 24, 48]. In addition, none of the studies made use of interspersed repeats, which account for 25–40% of the genomes of most higher organisms and allow genetic mutations to arise [64]. Based on these analogies, we are confident that the transcriptome data from PacBio Iso-Seq are more reliable in *S. europaea*. However, we suggest that further studies should be conducted in other organisms.

Global identification of *XTH* genes will help to understand gene expression and regulatory mechanisms for plant tolerance to environmental stresses such as salinity and drought. To date, many *XTH* genes have been identified in plants, including 33 in *A. thaliana* [39], 29 in *O. sativa* [37], 25 genes in *S. lycopersicum* [43], 56 in *N. tabacum* [17], 61 in *G. max* [36] and *H. vulgare* [38]. In this study, 35 *XTH* genes encoding the XTH domain were detected from *S. europaea*. In comparison, the number of *SeXTH* genes identified was slightly higher than that of *A. thaliana* (33), *O. sativa* (29), *S. lycopersicum* (25), and *H. vulgare* (24), suggesting that this might be a result of gene gains and losses [35]. *SeXTH* genes were categorized into three clusters: 29 genes formed group I/II, 3 were classified into group IIIA, and also 3 were classified into group IIIB, as described previously [62]. Previous studies reported that there was no significant divergence between group I and group II, which formed the largest cluster and is referred to as group I/II [38, 39]. So, this might be the reason that group I and group II were clustered together in this study (Fig. 1) and also had the largest members. Although group IIIB had obvious XET activity, group IIIA had significant XEH activity [26, 63], confirming a functional distinction between subgroup IIIA and IIIB. Therefore, we speculated that *SeXTH12, − 13,* and *− 22* in group IIIB might possess XET activities and *SeXTH25, − 28,* and *− 29* in group IIIA might exert XEH activity in *S. europaea* under abiotic stress. Further studies are required to fully elucidate the absence of an ancestral group in the *XTH* gene family of *S. europaea*.

XTH proteins have been found to have several conserved modular structures, including a short hydrophobic amino region that likely serves as a signal peptide to direct the protein to the plant cell wall and a highly conserved DEIDFEFLG domain that serves as a catalytic site for both XET and XEH activity [65]. In this study, a highly conserved XTH domain DEIDFEFLG was detected in almost all SeXTHs and an N-linked glycosylation site N(T)V(R/L/T/I)T(S/K/R/F/P)G was discovered near the catalytic residues (Fig. 2). However, it was discovered that the third amino acid, isoleucine (I), can also be replaced by alternative hydrophobic residues; either leucine (L), phenylalanine (F) or valine (V), and finally the fifth amino acid phenylalanine (F) can be replaced by isoleucine (I). This phenomenon has also been reported when comparing some catalytic domains of Arabidopsis XTHs [39, 65]. In this study, we discovered that the fifth phenylalanine (F) residue of SeXTH25 was replaced by methionine (M). Since the apolar and uncharged structure of the residues is preserved, Fu et al. [38] anticipated that these modifications would have little effect on the cleavage of the xyloglucan-glycan chain bonds. Interestingly, all ten (10) different types of motifs were conserved in *S. europaea.* Although the functions of the SeXTH motifs remain unclear, it is quite convincing that all the different motifs present in the SeXTH protein suggest that they might have different biochemical and biological functions under environmental stress.

Molecular control mechanisms for abiotic stress tolerance rely on the activation and regulation of specific stress-related genes. These genes are involved in the entire sequence of stress responses such as signal transduction, transcriptional control, membrane and protein protection [2]. The expression of *XTHs* varies upon stress exposure and exhibits tissue, organ, and time specificity [66]. In this study, the pattern of *SeXTHs* showed differential expression under different tissues, time, and NaCl treatments. For example, *SeXTH10/11* within group I/II was mainly expressed in roots but not in other tissues. The same phenomenon was reported in *O. sativa*, where seven *XTH* genes (*OsXTH1, − 2, − 4, − 13, − 15, − 16,* and *− 25*) were expressed mainly in roots of 14-day-old seedlings, whiles no expression was detected in other tissues [37]. *SeXTH17* was also found to be significantly expressed in shoots. A similar phenomenon was reported by Rose et al. [28], where *AtXTH24* was strongly expressed in shoots of *A. thaliana*. When the plantlets were exposed to 0, 50, and 200 mM NaCl, 27 and 15 *SeXTH* genes were highly expressed in shoots and roots, respectively, after 24 h at 200 mM NaCl. Similarly, *MtXTH3* gene was significantly up-regulated in the shoots and roots of *M. truncatula* when NaCl stress exceeded 150 mM NaCl; *CaXTH3* was also shown to increase salt tolerance in transgenic *A. thaliana* plants [35, 43]. Thus, these results suggest that several *SeXTH* genes are involved in high salt resistance that is tissue specific and functional redundancy based on expression patterns may not exist.

To better understand the possible function of *SeXTH* genes under drought stress, we also analyzed in detail the expression of *XTH* genes in *S. europaea*. A previous study reported that the expression of the *CaXTH3* gene from *C. annuum* was up-regulated when seedlings were exposed to extreme drought to improve drought resistance in transgenic *A. thaliana* [40] and *S. lycopersicum* [43]. In this work, rigorous expression analysis also showed that under 48 h of drought, 21 and 13 *SeXTH* genes were up-regulated in roots and shoots, respectively. Therefore, it would be reasonable to assume that the 34 *SeXTH* genes in *S. europaea* could be functional during the adaptation process to abiotic stress responses. In shoots, *SeXTH4/5, − 7/8, − 9, − 31,* and *− 32* were down-regulated at the onset of drought stress, which is consistent with the expression of several *XTH* isoform genes (*AtXTH6*, − *9, − 15, and − 16)* in *Arabidopsis* during mild and severe drought stress [66]. Also, Wu et al. [67] reported that *XTH* activity was reduced in the elongation region of soybean seedlings under low water potential. There were different expression patterns in both roots and shoots depending on the duration of drought stress. According to Tenhaken [68], different expression patterns of *XTH* genes were also found in shoots and roots of *Arabidopsis* plants subjected to 24 h drought stress. All the reported results clearly indicate that the expression of *SeXTH* genes can be an effective tool to improve the stress tolerance of some plants.

## Conclusion

In this study, 24,869 high-quality unigenes were generated by PacBio sequencing and 98% of these unigenes were predicted by CDSs. Based on this study, the transcriptome data from PacBio sequencing are more reliable in *S. europaea*. In this current study, 35 SeXTH proteins from *S. europaea* were discovered using bioinformatics. Based on their sequence conservation, the SeXTH proteins could be classified into three groups: Group I/II, Group IIIA and IIB according to their phylogenetic relationship. All showed the conserved domain DE(I/L/F/V)DF(I/M)EFLG with a potential N-linked glycosylation domain N(T)V(R/L/T/I)T(S/K/R/F/P)G. Expression analysis confirmed that *SeXTH* genes can help plants resist environmental stress.

## Supplementary Information

**Additional file 1: Supplementary file 1.** XTH sequences from *A. thaliana*, *N. tabacum* and *M. truncatula* used for phylogenetic tree construction.

**Additional file 2: Supplementary file 2.** The primer sequences used for the RT-qPCR analysis.

**Additional file 3: Supplementary file 3.** The RT-qPCR raw data.

**Additional file 4: Supplementary file 4.** Amino acid sequence of the predicted 35 SeXTH proteins.

**Additional file 5: Supplementary file 5.** The HMMER validation of the number of *SeXTH* genes present in *S. europaea*.

## Data Availability

All raw sequence data have been submitted to the Sequence Read Archive (SRA) database under accession number PRJNA725943 (https://submit.ncbi.nlm.nih.gov/subs/sra).

## Abbreviations

XTH: Xyloglucan endotransglycosylase/hydrolase
GH16: Glycoside hydrolase 16
GH16-XET: GH16 domain of XET
C-XET: C-terminal domain of XET
XEH: Xyloglucan endo-hydrolase
XET: Xyloglucan endo-transglycosylase
RNA-Seq: RNA sequencing
Iso-Seq: Isoform sequencing
CCS: Circular consensus sequences
CDSs: Coding sequences
NR: Non-redundant
KEGG: Kyoto Encyclopedia of Genes and Genomes
TFs: Transcription factors
MEME: Multiple expectation maximization for motif elicitation
RT-qPCR: Real-time quantitative polymerase chain reaction
UBC: Ubiquitin-conjugating
SRA: Sequence read archive
