# Supplementary material for: Transcriptome-wide characterization and functional analysis of Xyloglucan endo-transglycosylase/hydrolase (XTH) gene family of Salicornia europaea L. under salinity and drought stress
Source: BMC Plant Biol. 2021 Oct 25;21:491. doi: 10.1186/s12870-021-03269-y (PMC8547092; doi:10.1186/s12870-021-03269-y)
Supplement: Supplementary file 1 — Additional file 1: Supplementary file 1. XTH sequences from A. thaliana, N. tabacum and M. truncatula used for phylogenetic tree construction. [file 12870_2021_3269_MOESM1_ESM.docx]

Supplementary file 1: XTH sequences from *A. thaliana*, *N. tabacum* and *M. truncatula* used for phylogenetic tree construction.

>AtXTH1 MNKMEYLSIFGFVSVLYLIIRVDARAYEVNGIDQSKVGFDDNYVVTWGQNNVLKLNQGKEVQLSLDHSSGSGFESKNHYESGFFQIRIKVPPKDTSGVVTAFYLTSKGNTHDEVDFEFLGNKEGKLAVQTNVFTNGKGNREQKLALWFDPSKDFHTYAILWNPYQIVLYVDNIPVRVFKNTTSQGMNYPSKPMQVVVSLWNGENWATDGGKSKINWSLAPFKANFQGFNNSGCFTNAEKNACGSSAYWWNTGSYSKLSDSEQKAYTNVRQKYMNYDYCSDKVRFHVPPSECKWNN

>AtXTH2 MNRIRYCFELVSVLFLMFTANARARGRGAIDFDVNYVVTWGQDHILKLNQGKEVQLSMDYSSGSGFESKSHYGSGFFQMRIKLPPRDSAGVVTAFYLTSKGDTHDEVDFEFLGNRQGKPIAIQTNVFSNGQGGREQKFVPWFDPTTSFHTYGILWNPYQIVFYVDKVPIRVFKNIKKSGVNYPSKPMQLVASLWNGENWATSGGKEKINWAYAPFKAQYQGFSDHGCHVNGQSNNANVCGSTRYWWNTRTYSQLSANEQKVMENVRAKYMTYDYCSDRPRYPVPPSECRWNQ

>AtXTH3 MDYMRIFSVFVVTLWIIRVDARVFGGRGIEKFVTFGQNYIVTWGQSHVSTLHSGEEVDLYMDQSSGGGFESKDAYGSGLFEMRIKVPSGNTGGIVTAFYLTSKGGGHDEIDFEFLGNNNGKPVTLQTNLFLNGEGNREERFLLWFNPTKHYHTYGLLWNPYQIVFYVDNIPIRVYKNENGVSYPSKPMQVEASLWNGDDWATDGGRTKVNWSYSPFIAHFRDFALSGCNIDGRSNNVGACESSNYWWNAGNYQRLSGNEQKLYEHVRSKYMNYDYCTDRSKYQTPPRECY

>AtXTH4

MTVSSSPWALMALFLMVSSTMVMAIPPRKAIDVPFGRNYVPTWAFDHQKQFNGGSELQLILDKYTGTGFQSKGSYLFGHFSMHIKLPAGDTAGVVTAFYLSSTNNEHDEIDFEFLGNRTGQPAILQTNVFTGGKGNREQRIYLWFDPSKAYHTYSILWNMYQIVFFVDNIPIRTFKNAKDLGVRFPFNQPMKLYSSLWNADDWATRGGLEKTNWANAPFVASYKGFHIDGCQASVEAKYCATQGRMWWDQKEFRDLDAEQWRRLKWVRMKWTIYNYCTDRTRFPVMPAECKRDRDA

>AtXTH5

MGRLSSTLCLTFLILATVAFGVPPKKSINVPFGRNYFPTWAFDHIKYLNGGSEVHLVLDKYTGTGFQSKGSYLFGHFSMHIKMVAGDSAGTVTAFYLSSQNSEHDEIDFEFLGNRTGQPYILQTNVFTGGAGNREQRINLWFDPSKDYHSYSVLWNMYQIVFFVDDVPIRVFKNSKDVGVKFPFNQPMKIYSSLWNADDWATRGGLEKTNWEKAPFVASYRGFHVDGCEASVNAKFCETQGKRWWDQKEFQDLDANQYKRLKWVRKRYTIYNYCTDRVRFPVPPPECRRDRDI

>AtXTH6 MAKIYSPSFPGTLCLCIFTLLTLMFIRVSARPATFVEDFKAAWSESHIRQMEDGKAIQLVLDQSTGCGFASKRKYLFGRVSMKIKLIPGDSAGTVTAFYMNSDTATVRDELDFEFLGNRSGQPYSVQTNIFAHGKGDREQRVNLWFDPSMDYHTYTILWSHKHIVFYVDDVPIREYKNNEAKNIAYPTSQPMGVYSTLWEADDWATRGGLEKIDWSKAPFYAYYKDFDIEGCPVPGPTFCPSNPHNWWEGYAYQSLNAVEARRYRWVRVNHMVYDYCTDRSRFPVPPPECRA

>AtXTH7 MVVSLFSSRNVFYTLSLCLFAALYQPVMSRPAKFEDDFRIAWSDTHITQIDGGRAIQLKLDPSSGCGFASKKQYLFGRVSMKIKLIPGDSAGTVTAFYMNSDTDSVRDELDFEFLGNRSGQPYTVQTNVFAHGKGDREQRVNLWFDPSRDFHEYAISWNHLRIVFYVDNVPIRVYKNNEARKVPYPRFQPMGVYSTLWEADDWATRGGIEKINWSRAPFYAYYKDFDIEGCPVPGPADCPANSKNWWEGSAYHQLSPVEARSYRWVRVNHMVYDYCTDKSRFPVPPPECSAGI

>AtXTH8 METERRIITSCSAMTALFLFMTALMASSSIAATPTQSFEDNFNIMWSENHFTTSDDGEIWNLSLDNDTGCGFQTKHMYRFGWFSMKLKLVGGDSAGVVTAYYMCSENGAGPERDEIDFEFLGNRTGQPYIIQTNVYKNGTGNREMRHSLWFDPTKDYHTYSILWNNHQLVFFVDRVPIRVYKNSDKVPNNDFFPNQKPMYLFSSIWNADDWATRGGLEKTDWKKAPFVSSYKDFAVEGCRWKDPFPACVSTTTENWWDQYDAWHLSKTQKMDYAWVQRNLVVYDYCKDSERFPTLPWECSISPWA

>AtXTH9 MVGMDLFKCVMMIMVLVVSCGEAVSGAKFDELYRSSWAMDHCVNEGEVTKLKLDNYSGAGFESRSKYLFGKVSIQIKLVEGDSAGTVTAFYMSSDGPNHNEFDFEFLGNTTGEPYIVQTNIYVNGVGNREQRLNLWFDPTTEFHTYSILWSKRSVVFMVDETPIRVQKNLEEKGIPFAKDQAMGVYSSIWNADDWATQGGLVKTDWSHAPFVASYKEFQIDACEIPTTTDLSKCNGDQKFWWDEPTVSELSLHQNHQLIWVRANHMIYDYCFDATRFPVTPLECQHHRHL

>AtXTH10 MTLINRSKPFVLLVGFSIISSLLLWVSQASVVSSGDFNKDFFVTWSPTHVNTSNDGRSRTLKLDQESGASFSSIQTFLFGQIDMKIKLIRGSSQGTVVAYYMSSDQPNRDEIDFEFLGNVNGQPYILQTNVYAEGLDNREERIHLWFDPAKDFHTYSILWNIHQIVFMVDQIPIRLYRNHGEKGVAYPRLQPMSVQASLWNGESWATRGGHDKIDWSKGPFVASFGDYKIDACIWIGNTSFCNGESTENWWNKNEFSSLTRVQKRWFKWVRKYHLIYDYCQDYGRFNNKLPKECSLPKY

>AtXTH11 MRGSDQKILLMVMVVVAVVAAAQGQEETTGFVTWGNNYYQTWGHQALVINKTSELQLTLDKNSGSGFESQLIYGSGYFNVRIKAPQTTSTGVITSFYLISRSSRHDELCFQILGKNGPPYLLNTNMYLYGEGGKDQRFRLWFYVDDTPIRVYSKNPDVYYPSVQTMFLMGSVQNGSIIDPKQMPYIAKFQASKIEGCKTEFMGIDKCTDPKFWWNRKQLSSKEKTLYLNARKTYLDYDYCSDRQRYPKVPQECGSYT

>AtXTH12 MAAFATKQSPLLLASLLILIGVATGSFYDSFDITWGAGRANIFESGQLLTCTLDKTSGSGFQSKKEYLFGKIDMKIKLVPGNSAGTVTAYYLSSKGETWDEIDFEFLGNVTGQPYVIHTNVFTGGKGNREMQFYLWFDPTADFHTYTVLWNPLNIIFLVDGIPIRVFKNNEANGVAYPKSQPMKIYSSLWEADDWATQGGKVKTDWTNAPFSASYRSFNDVDCCSRTSIWNWVTCNANSNSWMWTTLNSNQLGQLKWVQKDYMIYNYCTDFKRFPQGLPTECNLN

>AtXTH13

MAAFTTKQSLLLLSLLLLISLSAGSFYDNFDITWGNGRANIVESGQLLTCTLDKISGSGFQSKKEYLFGKIDMKMKLVAGNSAGTVTAYYLSSKGETWDEIDFEFLGNVTGQPYVLHTNVFTGGKGNREMQFYLWFDPTADFHTYTVLWNPLNIIFLVDGIPIRVFKNNEANGVAYPKSQPMKIYSSLWEADDWATQGGKVKTDWTNAPFSASYKSFNDVDCCSRTSLLNWVTCNANSNSWMWTTLNSNQYGQMKWVQDDYMIYNYCTDFKRFPQGLPTECNLN

>AtXTH15 MGPSSSLTTIVATVLLVTLFGSAYASNFFDEFDLTWGDHRGKIFNGGNMLSLSLDQVSGSGFKSKKEYLFGRIDMQLKLVAGNSAGTVTAYYLSSQGATHDEIDFEFLGNETGKPYVLHTNVFAQGKGDREQQFYLWFDPTKNFHTYSIVWRPQHIIFLVDNLPIRVFNNAEKLGVPFPKSQPMRIYSSLWNADDWATRGGLVKTDWSKAPFTAYYRGFNAAACTASSGCDPKFKSSFGDGKLQVATELNAYGRRRLRWVQKYFMIYNYCSDLKRFPRGFPPECKKSRV

>AtXTH16 MGRILNRTVLMTLLVVTMAGTAFSGSFNEEFDLTWGEHRGKIFSGGKMLSLSLDRVSGSGFKSKKEYLFGRIDMQLKLVAGNSAGTVTAYYLSSEGPTHDEIDFEFLGNETGKPYVLHTNVFAQGKGNREQQFYLWFDPTKNFHTYSLVWRPQHIIFMVDNVPIRVFNNAEQLGVPFPKNQPMKIYSSLWNADDWATRGGLVKTDWSKAPFTAYYRGFNAAACTVSSGSSFCDPKFKSSFTNGESQVANELNAYGRRRLRWVQKYFMIYDYCSDLKRFPQGFPPECRKSRV

>AtXTH17 MKLSCGTSFAFLLLFLLAAQSVHVYAGSFHKDVQIHWGDGRGKIHDRDGKLLSLSLDKSSGSGFQSNQEFLYGKAEVQMKLVPGNSAGTVTTFYLKSPGTTWDEIDFEFLGNISGHPYTLHTNVYTKGTGDKEQQFHLWFDPTVNFHTYCITWNPQRIIFTVDGIPIREFKNPEAIGVPFPTRQPMRLYASLWEAEHWATRGGLEKTDWSKAPFTAFYRNYNVDGCVWANGKSSCSANSPWFTQKLDSNGQTRMKGVQSKYMIYNYCTDKRRFPRGVPAECT

>AtXTH18 MKLSCGTSFAFLIMFLFAAQSMHVYAGSFHKDVQIHWGDGRGKVRDRDGKLLSLSLDKSSGSGFQSNQEFLYGKAEVQMKLVPGNSAGTVTTFYLKSPGTTWDEIDFEFLGNLSGHPYTLHTNVYTKGSGDKEQQFHLWFDPTVNFHTYCITWNPQRIIFTVDGIPIREFKNSESIGVPFPTKQPMRLYASLWEAEHWATRGGLEKTDWSKAPFTAFYRNYNVEGCVWANGKSSCPANSSWFTQQLDSNGQTRMKGVQSKYMVYNYCNDKRRFPRGVPVECS

>AtXTH19 MKSFTFLILFLFAAQSISVYAGSFHKDVKIHWGDGRGKIHDNQGKLLSLSLDKSSGSGFQSNQEFLYGKAEVQMKLVPGNSAGTVTTFYLKSPGTTWDEIDFEFLGNISGHPYTLHTNVYTKGSGDKEQQFHLWFDPTANFHTYCITWNPQRIIFTVDGIPIREFMNAESRGVPFPTKQPMRLYASLWEAEHWATRGGLEKTDWSKAPFTAYYRNYNVEGCVWVNGKSVCPANSQWFTQKLDSNGQTRMKGVQSKYMVYNYCSDKKRFPRGVPPECS

>AtXTH20 MVSFCGRRFAFLIIFLFAAQYERVYAGSFHKDVQIHWGDGRGKILDNVGNLLSLSLDKFSGSGFQSHQEFLYGKVEVQMKLVPGNSAGTVTTFYLKSPGTTWDEIDFEFLGNISGHPYTLHTNVYTKGTGDKEQQFHLWFDPTVDFHTYCIIWNPQRVIFTIDGIPIREFKNSEALGVPFPKHQPMRLYASLWEAEHWATRGGLEKTDWSKAPFTAFYRNYNVDACVWSNGKSSCSANSSWFTQVLDFKGKNRVKWAQRKYMVYNYCTDKKRFPQGAPPECS

>AtXTH21 MVSSTLLVMSISLFLGLSILLVVHGKDFNQDIDITWGDGRGNILNNGTLLNLGLDQSSGSGFQSKAEYLYGKVDMQIKLVPGNSAGTVTTFYLKSQGLTWDEIDFEFLGNVSGDPYIVHTNVYTQGKGDREQQFYLWFDPTAAFHNYSILWNPSHIVFYIDGKPIREFKNLEVLGVAYPKNQPMRMYGSLWNADDWATRGGLVKTNWSQGPFVASFMNYNSENACVWSIVNGTTTTSPCSPGDSTSSSSSSTSEWFSQRGMDSSSKKVLRWVQRKFMVYNYCKDKKRFSNGLPVECTAKNKNTKS

>AtXTH22

MAITYLLPLFLSLIITSSVSANFQRDVEITWGDGRGQIKNNGELLTLSLDKSSGSGFQSKNEYLFGKVSMQMKLVPGNSAGTVTTLYLKSPGTTWDEIDFEFLGNSSGEPYTLHTNVYTQGKGDKEQQFKLWFDPTANFHTYTILWNPQRIIFTVDGTPIREFKNMESLGTLFPKNKPMRMYSSLWNADDWATRGGLVKTDWSKAPFTASYRGFQQEACVWSNGKSSCPNASKQGTTTGSWLSQELDSTAQQRMRWVQRNYMIYNYCTDAKRFPQGLPKECLAA

>AtXTH23

MAMISYSTIVVALLASFMICSVSANFQRDVEITWGDGRGQITNNGDLLTLSLDKASGSGFQSKNEYLFGKIDMQIKLVAGNSAGTVTAYYLKSPGSTWDEIDFEFLGNLSGDPYTLHTNVFTQGKGDREQQFKLWFDPTSDFHTYSILWNPQRIIFSVDGTPIREFKNMESQGTLFPKNQPMRMYSSLWNAEEWATRGGLVKTDWSKAPFTASYRGFNEEACVVINGQSSCPNVSGQGSTGSWLSQELDSTGQEQMRWVQNNYMIYNYCTDAKRFPQGLPRECLAA

>AtXTH24 MSPFKIFFFTTLLVAAFSVSAADFNTDVNVAWGNGRGKILNNGQLLTLSLDKSSGSGFQSKTEYLFGKIDMQIKLVPGNSAGTVTTFYLKSEGSTWDEIDFEFLGNMSGDPYTLHTNVYTQGKGDKEQQFHLWFDPTANFHTYSILWNPQRIILTVDDTPIREFKNYESLGVLFPKNKPMRMYASLWNADDWATRGGLVKTDWSKAPFMASYRNIKIDSKPNSNWYTQEMDSTSQARLKWVQKNYMIYNYCTDHRRFPQGAPKECTTSS

>AtXTH25

MDRSTFILSLLFTLTVSTTTLFSPVFAGTFDTEFDITWGDGRGKVLNNGELLTLSLDRASGSGFQTKKEYLFGKIDMQLKLVPGNSAGTVTAYYLKSKGDTWDEIDFEFLGNLTGDPYTMHTNVYTQGKGDREQQFHLWFDPTADFHTYSVLWNPHHIVFMVDDIPVREFKNLQHMGIQYPKLQPMRLYSSLWNADQWATRGGLVKTDWSKAPFTASYRNFRADACVSSGGRSSCPAGSPRWFSQRLDLTAEDKMRVVQRKYMIYNYCTDTKRFPQGFPKECRH

>AtXTH26 MAGLQAKTLMFVLAAALATLGRTFVEADFSKNFIVTWGKDHMFMNGTNLRLVLDKSAGSAIKSKVAHLFGSVEMLIKLVPGNSAGTVAAYYLSSTGSTHDEIDFEFLGNATGQPYTIHTNLYAQGKGNREQQFRPWFNPTNGFHNYTIHWNPSEVVWFVDGTPIRVFRNYESEGIAYPNKQGMKVFASLWNAEDWATQGGRVKTNWTLAPFVAEGRRYKARACLWKGSVSIKQCVDPTIRSNWWTSPSFSQLTASQLTKMQKIRDGFMIYDYCKDTNRFKGVMPPECSKKQF

>AtXTH27

METLSRLLVFMSLFSGLVSGFALQNLPITSFEESYTQLFGDKNLFVHQDGKSVRLTLDERTGSGFVSNDYYLHGFFSASIKLPSDYTAGVVVAFYMSNGDMYEKNHDEIDFEFLGNIREKEWRVQTNIYGNGSTHSGREERYNLWFDPTEDFHQYSILWSDSHIIFFVDNVPIREVKRTAEMGGHFPSKPMSLYTTIWDGSKWATNGGKYGVNYKYAPYIARFSDLVLHGCPVDPIEQFPRCDEGAAEDMRAAQEITPSQRSKMDVFRRRLMTYSYCYDRARYNVALSECVVNPAEAQRLRVYDPVRFGGIPRRHRNGKHRSKRSRVDGTESI

>AtXTH28 MGFITRFLVFMSLFTSLVSGFALQKLPLIQFDEGYTQLFGDQNLIVHRDGKSVRLTLDERTGSGFVSNDIYLHGFFSSSIKLPADYSAGVVIAFYLSNGDLYEKNHDEIDFEFLGNIRGREWRIQTNIYGNGSTHLGREERYNLWFDPTEDFHQYSILWSLSHIIFYVDNVPIREVKRTASMGGDFPAKPMSLYSTIWDGSKWATDGGKYGVNYKYAPYVSQFTDLILHGCAVDPTEKFPSCKDEAVQNLRLASEITESQRNKMEIFRQKHMTYSYCYDHMRYKVVLSECVVNPAEAKRLRVYDPVTFGGIPHGHRRGKHRSRSRLARTESI

>AtXTH29 MRDSIYLLWIDNRLVVIIMMVMMVSCRCVLGLENINPIFFDEGLSHLFGEGNLIRSPDDRSVRLLLDKYTGSGFISSSMYQHGFFSSLIKLPGAYTAGIVVAFYTSNGDVFVKDHDELDIEFLGNLEGKPWRFQTNMYGNGSTNRGREERYRLWFDPSKEFHRYSILWTPTKIIFWVDDVPIREILRKEEMNGDYPQKPMSLYATIWDASSWATSGGKFGVDYTFSPFVSEFKDIALDGCNVSDSFPGENNNNNIGNYNNINCSVSDQFLMSNDYSTISPKQATAMRRFRERYMYYSYCYDTIRYSVPPPECVIVTAEKNRFRDTGRLKFGGSHPKVHKARKKRRRNRSTPVVSADL

>AtXTH30 MSKSSYNHIFILILCLCLRSSSAFTNLNTLSFEESLSPLFGDANLVRSPDDLSVRLLLDRYTGSGFISSNMYQHGFYSSMIKLPADYTAGVVVAFYTSNGDVFEKTHDELDIEFLGNIKGKPWRFQTNLYGNGSTHRGREERYRLWFDPSKEFHRYSILWTPHKIIFWVDDVPIREVIRNDAMGADYPAKPMALYATIWDASDWATSGGKYKANYKFAPFVAEFKSFSLDGCSVDPIQEVPMDCSDSVDFLESQDYSSINSHQRAAMRRFRQRFMYYSYCYDTLRYPEPLPECVIVPAEKDRFKETGRLKFGGTEARERRRNRRQQRRPEIEIESDPDDRKLL

>AtXTH31

MALSLIFLALLVLCPSSGHSQRSPSPGYYPSSRVPTSPFDREFRTLWGSQHQRREQDVVTLWLDKSTGSGFKSLRPYRSGYFGASIKLQPGFTAGVDTSLYLSNNQEHPGDHDEVDIEFLGTTPGKPYSLQTNVFVRGSGDRNVIGREMKFTLWFDPTQDFHHYAILWNPNQIVFFVDDVPIRTYNRKNEAIFPTRPMWVYGSIWDASDWATENGRIKADYRYQPFVAKYKNFKLAGCTADSSSSCRPPSPAPMRNRGLSRQQMAALTWAQRNFLVYNYCHDPKRDHTQTPEC

>AtXTH32

MGNSLISLLSIFHLLVLWGSSVNAYWPPSPGYWPSSKVGSLNFYKGFRNLWGPQHQRMDQNALTIWLDRTSGSGFKSVKPFRSGYFGANIKLQPGYTAGVITSLYLSNNEAHPGFHDEVDIEFLGTTFGKPYTLQTNVYIRGSGDGKIIGREMKFRLWFDPTKDFHHYAILWSPREIIFLVDDIPIRRYPKKSASTFPLRPMWLYGSIWDASSWATEDGKYKADYKYQPFTAKYTNFKALGCTAYSSARCYPLSASPYRSGGLTRQQHQAMRWVQTHSMVYNYCKDYKRDHSLTPECWR

>AtXTH33

MKIMWETAVVFCLCSLSLVSSHSRKFTTPNVTRLTDQFSKIAIENGFSRRFGAHNIQVNGSLAKLTLDKSSGAGLVSKNKYHYGFFSARLKLPAGFASGVVVAFYLSNAETYPKSHDEIDIELLGRSRRDDWTIQTNVYANGSTRTGREEKFYFWFDPTQAFHDYTLIWNSHHTVFLVDNIPVRQFPNRGAFTSAYPSKPMSLYVTVWDGSEWATKGGKYPVNYKYAPFVVSVADVELSGCSVNNGSSTGSGPCTKSGGSISSLDPVDGQDFATLSKNQINAMDWARRKLMFYSYCSDKPRYKVMPAECN

>Medtr4g128410.1

MAFSKFVTFLVIFISTVAIASAGNFYQDFDITWGDGRAKILNSGQLLTLSLDKSSGSGFQSKNEYLFGKIDMQLKLVPGNSAGTVTAYYLSSKGGAWDEIDFEFLGNLSGDPYILHTNVFSQGKGDREQQIYLWFDPTADFHTYSILWNPQRIVFSVDGTPIREFKNIESSGVPFPKNQPMRIYSSLWNADDWATRGGLVKTDWSKAPFTASYRHFNANACIVSSGISSCDSAQSNKNDWFSEELDSTSYERLKWVQKNYMIYNYCSDNKRFPQGLPQECRMT

>Medtr7g093530.1

MAFSRVTFLVILISTVAIASAGNFYQDFDITWGDGRAKILNNGQLLTLSLDKSSGSGFQSKNEYLFGKIDMQLKLVPGNSAGTVTAYYLSSKGGAWDEIDFEFLGNLSGDPYILHTNVFSQGKGNREQQFYLWFDPTADFHTYSILWNPQRIVFSVDGSPIREFKNMESNGVPFPKSQPMRIYSSLWNADDWATRGGLVKTDWSNAPFTASYRNFNANACTVSSGTSSCSSTQSNKNSWFSEELDSTSHERLKWVQKNYMIYNYCTDTKRFPQGLPQECMMV

>Medtr4g128570.1

MAYTHNCSMITVLLSLLVFATFMTTYAAGNFYQNFDITWGDGRAKILDNGQLLTLSLDKASGSGFQSKNEYLFGNIDMQLKLVPGNSAGTVTAYYLSSKGSNWDEIDFEFLGNVSGEPYTLHTNVFCQGKGNREQQFHLWFDPTADFHTYSILWNPQRIVFSVDGTPIREFKNMESKGVAFPKNQPMRIYSSLWNADDWATRGGLVKTDWTNAPFTASYRNFNAETTSSNAWFTQQLDSTSQQRLSEVQKNYMIYNYCTDIKRFPQGLPTECTAS

>Medtr4g128580.1

MASTTTLLVLLIPLLWINVACVAGNFNKDFQITWGDGRAKILNNANLLTLSLDKASGSGFQSKNEYLFGKIDMQIKLVPGNSAGTVTAYYLSSKGGAWDEIDFEFLGNLSGDPYILHTNVFSQGKGNREQQFYLWFDPTADFHTYSILWNPQRIVFSVDGTPIREFKNMESNGVPFPKNQPMRIYSSLWNADDWATRGGLVKTDWSKAPFTASYRNFNANNACIWNGGKSSCKSSSKPSASSASWLSQQLDSTGQQRLRWVQKNYMIYNYCSDKKRFPQGLPVECTHS

>Medtr4g128590.1

MVSSNDFNMLILIPLLVSSLMVMNAFAAGNLNQHFDITWGDGRAKILNNGELLTLSLDKIDMQLKLVPGNSAGTVTAYYLSSKGPTWDEIDYEFLGNVSGEPYILHTNVFSQGKGNREQQFYLWFDPAADFHTYSIIWNPQRIIFSVDGTPIREFKNSETIGVPFPKNQPMRIYSSLWNADNWATRGGLVKTDWTKAPFTASYRNFNAQACIWSSGASSCGSTSSASKDSARGSWLSQELDATGQGRLKWVQKNYMIYNYCKDTKRFPQGLPPECNHS

>Medtr4g128400.1

MASNYAFFVHVITSLICTIFIVAFAGNLYQDVGITWGDGRGKIVNNGQLLTLSLDRTSGSGFQSNNQYLYGKIDMQIKLVPGNSAGTVTAYYLRSEGSLWDEIDFEFLGNLSGDPYIVHTNVYTQGKGDREQQFYLWFDPTTSFHTYSFLWNPAHVVFSIDGRPIREFKNLESEGVPYPKKQPMRLYSSLWNADDWATRGGLVKTDWNQAPFTASFRNFKANGCVLSNGISSCKSNSSSDNAWLYQQLDSTNQKRLKWVQKNYMIYNYCNDLKRFPQGLPLECIVRTNS

>Medtr4g126920.1

MTNKIILINLLLITFLVEASAASNFNQYFDITWGDGRGKILNNGQLLTLSLDKASGSGFRSKNEYLFGKIDMQLKLVPGNSAGTVTTYYLSSLGDAHDEIDFEFLGNLSGDPYIVHTNIYAQGKGNKEQQFYLWFDPTKDFHTYSILWNPQSIIFSVDGTPLREFKNLESKGIPFPKSQAMSIYSSLWDADNWATRGGLAKTDWSQAPFTASYGNFNIQACVWTSSGSSCSSKNPSSNHSWMKQSLDSTGQARIQWVQKNYMIYNYCTDTKRFPQGLPPECSLA

>Medtr2g038705.1

MKFKGDIGLFLLLVATFLVYASAGNFMKDFEITWGESRAKVLENGQSLSLTLDKSSGSGFRSKYEYLFGKIDMQLKLVAGNSAGIVTAYYLSSLGSTHDEIDFEFLGNLSGDPYILHTNVFTQGKGNREQQFYLWFDPTKDFHTYSLRWNPKSIIFSVDGRPIREFKNLESKGVPFPKNQAMRIYSSLWNADNWATRGGIVKTDWTNAPFIASYRNFNAKACIWTSSGSSCSSNKSPLSSSTSQSWLRESLDSKGIRKIRWVQKNYMIYNYCTDYKRFPQGFPAECITSKPHKL

>Medtr2g038860.1

MKFKGDIGLFLLLVATFLVYASAGNFMKDFEITWGESRAKVLENGQSLSLTLDKSSGSGFRSKYEYLFGKIDMQLKLVAGNSAGIVTAYYLSSLGSTHDEIDFEFLGNLSGDPYILHTNVFTQGKGNREQQFYLWFDPTKDFHTYSLRWNPKSIIFSVDGRPIREFKNLESKGVPFPKNQAMRIYSSLWNADNWATRGGIVKTDWTNAPFIASYRNFNAKACIWTSSGSSCSSNKSPLSSSTSQSWLRESLDSKGIRKIRWVQKNYMIYNYCTDYKRFPQGFPAECITSKPHKL

>Medtr8g081040.1

MASNSTHNEFHVFMLIAIMVSSMVATCAGSFYQDFDLTWGDNRAKIFNGGQLLSLSLDKVSGSGFKSKREYLFGRIDMQLKLVAGNSAGTVTAYYLSSQGPTHDEIDFEFLGNTSGDPYILHTNIFTQGKGNREQQFYLWFDPTRNFHTYSIIWKPQHIIFLVDNMPIRVFKNVESIGVPFPKNQPMRIYSSLWNADDWATRGGLVKTDWSKAPFTAYYRNFKATQFSTKSSLSSNSDAEWQINELDAYGRRRLRWVQKYFMIYNYCNDLKRFPQGVPVECSH

>Medtr8g102220.1

MASSYIRMNCMLIGAMIVSSLIATCCANFYQDFDLTWGDHRAKTFNGGQLLSLSLDKVSGSGFQSKKEYLFGRIDMQLKLVAGNSAGTVTAYYLSSQGPTHDEIDFEFLGNVSGNPYILHTNVFSQGKGNREQQFYLWFDPTKNFHTYSIIWKPRRIIFLVDNIPIRIFKNAESISIPFPKNQPMRIYSSLWNAEDWATRGGLLTTAISKLLNYPVSSTSFSDSALQSNELDAYGRRRLRWVQKYFMIYNYCNDLKRFPQGIPAECRRSRTTIL

>Medtr3g084620.1

MAYSIGINSMLIGVMIISSLVATCSANFNQDFDLTWGDHRAKIFNGGQLLSLSLDKTSGSGFQSKKEYLFGRIDMQLKLVAGNSAGTVTAYYLSSQGPTHDEIDFEFLGNVSGDPYILHTNVFSQGKGNREQQFYLWFDPTKNFHTYSIIWKPQHIIFLVDNTPIRIFKNAESIGIPFPKNQPMRIYSSLWNADDWATRGGLVKTDWSKAPFTAYYRNFKATELSSVSSTSFSDSALQSNELDAYGRRRLRWVQKYFMIYNYCNDLKRFPEGIPAECKRPRF

>Medtr3g084630.1

MVSSIGINNMLIGVMIISSLVATCSANFNQDFDLTWGDHRAKIFNGGQLLSLSLDKTSGSGFQSKKEYLFGRIDMQLKLVAGNSAGTVTAYYLSSQGPTHDEIDFEFLGNVSGDPYILHTNVFSQGKGNREQQFYLWFDPTKNFHTYSIIWKPQHIIFLVDNTPIRIFKNAESIGIPFPKNQPMRIYSSLWNADDWATRGGLVKTDWSKAPFTAYYRNFKATELSSVSSTSFSDSALQSNELDAYGRRRLRWVQKYFMIYNYCNDLKRFPEGIPAECKRPRF

>Medtr7g084750.1

MEFSLWIRSLILLSLTSAALSATPRKPVDVPFGRNYVPTWAFDHIKYFNEGSEIELLLDKYTGTGFQSKGSYLFGHFSMNIKMVPGDSAGTVTAFYLSSQNADHDEIDFEFLGNRTGQPYILQTNVFTGGQGNREQRIFLWFDPTKAYHRYSVLWNMYQIVFFVDNIPIRVFKNSKKLGVKFPFDQPMKVYNSLWNADDWATRGGLEKTDWSKAPFIAGYKSFHIDGCETSVNAKYCAKQGRKWWNRPEFRDLDAAQWKRIKWVRKKFTIYNYCTDRTRFPQIPPECRRDRDI

>Medtr6g033055.1

MNSSLWNCLILLSSFVYASFAANPRTPIDVPFGRNYVPTWAYDHIKYLNGGSEILLNLDKSTGTGFQSKGSYLFGHFSMYIRMVAGDSAGTVTAFYLSSQTAEHDEIDFEFLGNRTGQPYILQTNVFTGGKGDREQRIYLWFDPTKAYHRYSVLWNMFQIVFFVDDIPIRVFKNINQLGVKYPFSQPMKIYNSLWNADDWATRGGLEKTDWSKAPFIASYKGFHIDGCEASVNAKFCDTQGKRWWDQPEFHDLDVAQWQRLRWVRQKYTIYNYCNDRKRYPQVPQECTRDRDI

>Medtr6g033085.1

MNYSLRMCLILLSSFVYASFAANPRTPIDVPFGRNYVPTWAYDHIKYLNGGSEILLNLDKSTGTGFQSKGSYLFGHFSMFIKMVPGDSAGTVTAFYLSSQTAEHDEIDFEFLGNRTGQPYILQTNVFTGGKGDREQRIYLWFDPTQAYHRYSVLWNMFQIVFFVDDIPIRVFKNTNQLGGKYPFNQPMKIYNSLWNADDWATRGGLEKTDWSKAPFIASYKGFHIDGCEASVNAKFCDTQGKRWWDQPEFHDLDVAQWQRLRWVRQKYTIYNYCNDRKRYPQVPLECTRDRDV

>Medtr7g084760.1

MSSSFWTLCLILASLVSFSLCAPPRTPVDVPFGRNYYPTWAFDHIKYFNGGSEIQLHLDKYTGTGFQSKGSYLFGHFSMNIKMVPGDSAGTVTAFYLSSQNAEHDEIDFEFLGNRTGQPYILQTNVFTGGQGNKEQRIFLWFDPTKEFHRYSILWNMYQIVFFVDDVPIRVFKNSKDLGVKFPFDQPMKIYNSLWNADDWATRGGLEKTDWSKAPFIAGYKGFHIDGCESSVNAKFCATQGKRWWDQPEFRDLDAAQWRRLRWVRQKYTIYNYCTDRKRLPQVPPECKRDQDI

>Medtr7g084770.1

MSSSFWSLCLILASLVSSSLCAPPRKPVDVPFGRNYYPTWAFDHIKYFNGGSEIQLHLDKYTGTGFQSKGSYLFGHFSMNIKMVPGDSAGTVTAFYLSSQNAEHDEIDFEFLGNRTGQPYILQTNVFTGGQGNKEQRIFLWFDPTKEFHRYSILWNMYQIVFFVDDVPIRVFKNSKDLGVKFPFDQPMKIYNSLWNADDWATRGGLEKTDWSKAPFIAGYKSFHIDGCESSVNAKFCATQGKRWWDQPEFRDLDAAQWRRLRWVRQKYTIYNYCTDRKRLPQIPPECKRDRDI

>Medtr6g088320.1

MENFEKLFILFLFALIHTIVLVDANFSKSMYITWGAQHAILQGEDLQLVLDKTSGSAAETKKRFLFGTIESRIKLVPGNSAGTVTAYYLSSIGSQHDELDFEFLGNSSGQPYIVHTNIYTQGNGSREQQFYLWFDPTADFHNYTIHWNPTEIVWYIDSIPIRVFRNYENEGIAYPNKQGMKVYTSLWNADNWATRGGLVKTNWTNAPFVAKFNHFRARACKWNGAISINQCALNIAANWWTSPTYKQLGYAQLGQMNWVRNNYMIYDYCRDTKRFNGQMPPECFKAQF

>Medtr8g102910.1

MLVAFFICALMITNIIQVNANFSKSMYLTWGAQHASIVGEDLHLVLDKTSGSAARSKRSFLFGSIEMLIKLIPGNSAGIVTAYYLSSTGSQHDEIDFEFLGNSTGQPYTVNTNLFTQGKGSREQQFHLWFDPTADFHNYTIHWNPTEIVWYVDSMPIRVFRNYEHEGIAYPNKQGMRVYTSLWNADNWATRGGLVKTDWSKAPFKVGFHHFRARACKWNGAASINQCASNVKANWWTSSVYKHLSYGKIRQLNWVKKNFMTYDYCKDYKRFNGHIPHECFKTQF

>Medtr1g105900.1

MNNFNTKLIFFFFGLVSSSLFHISIASIVSTGDFNKDFFVIWSPNHVNTSDDGKTRSLKLDQESGAGFASNQMFLFGQIDMQIKLVPGDSAGTVLAFYLTSDQPNRDEIDLEFLGNVSGQPYILQTNIYADGFDNREERIFLWFDPTKDFHTYSVLWNLHQIVFMVDTIPIRVYRNHADKGVAFPRWQPMSLKATLWNGDSWATRGGQDKIDWKNGPFIASFRNYKIDACVWKGNPRFCRAASSTNWWNQFNFSSLTSIQRRWFKWVRKYHMIYDYCQDNERFQNNLPKECSLPKY

>Medtr2g089140.2

MFGKVTVHLKLVEGDSAGTVTAFYMSSEGPNHNEFDFEFLGNTTGEPYSVQTNVYVNGVGNREQRLNLWFDPSKDFHTYSIFWNQRQVVFLVDETPIRVHTNLEHKGIPFPKDQAMGVYSSIWNADDWATQGGRVKTDWTHAPFIATYKDFEINACEVAVPVTSTENAKKCASSEDKKYWWDEPMLNELTIHQSHQLIWVRANHMVYDYCADTARFPAIPLECVRHHH

>Medtr2g089140.1

MTRMSCLLGLSLCFLFVGLVASSKFEELFQPGWAMDHFVHEGDLLKLKLDNYSGAGFQSKSKYMFGKVTVHLKLVEGDSAGTVTAFYMSSEGPNHNEFDFEFLGNTTGEPYSVQTNVYVNGVGNREQRLNLWFDPSKDFHTYSIFWNQRQVVFLVDETPIRVHTNLEHKGIPFPKDQAMGVYSSIWNADDWATQGGRVKTDWTHAPFIATYKDFEINACEVAVPVTSTENAKKCASSEDKKYWWDEPMLNELTIHQSHQLIWVRANHMVYDYCADTARFPAIPLECVRHHH

>Medtr4g045853.1

MSSKMCWFFSFFVCLMLSVSLSTSSKFDELFQPSWAFDHFLHDGDLLKLKLDNSSGAGFVSKSKYMFGRVSIQLKLVEGDSAGTVTAFYMSSEGPNHNEFDFEFLGNTTGEPYSVQTNVYVNGIGNREQRLNLWFDPTKDFHSYSIFWNQRQVVFLVDETPIRVHTNMEHRGIPYPKDQPMGVYSSIWNADDWATQGGRVKTDWSHAPFIATYKSFEINACECPISVAAMDNTKRCSSNEDKKYWWDEPNLSVLNLHQSHQLMWVRNHHMVYDYCSDGSRFPITPLECVHHHHS

>Medtr1g105495.1

MDGGSFSSFSILLFLAAIVAVYHHSAEAAMSKGSFEDNFSIMWSENHFTTSTDGQIWYLSLDNDTGCGFQTKQRYRFGWFSMKLKLVGGDSAGVVTAYYMCTENGAGPERDELDFEFLGNRTGQPYLIQTNVYKNGTGNREMRHMLWFDPTEDYHTYSILWNNHQIVFFVDRVPIRVFKNNDKPNNFFPNEKPMYLFSSVWNADDWATRGGLEKTNWKLAPFVSSYKDFSVDACQWEDPFPKCVSTTTKNWWDQYDAWHLSGDQKMDYAWVQRNLVIYDYCNDSERFPTLPEECSLSPWE

>Medtr8g479340.1

MCTENGAGPERDELDFEFLGNRTGQAYLIQTNVYKNELDRVWNADDWATRGGLEKTNWKLAPFVSSYKDFSVDACQWEDPFPKCVSTTTKNWWDQYDAWLLSGDQKMDYAWVQRNLVIYDYCNHSERFPTLPEECSLSPWE

>Medtr4g097670.1

MAIFYPFKNNGVFFMLLLWIVVSSVWGRPATFNQDFHVTWSEPHIKQIDQGRTIQLTLDQGSGCGFASKVKYLFGRVSMKIKLVPGDSAGTVTAFYMNSDTDSVRDELDFEFLGNRTGQPYTVQTNIYAHGKGDREQRVNLWFDPSADFHTYSILWNHHHIVFYVDEVPIRVYKNNEAKGIPYPKMQAMGVFSTLWEADNWATRGGLEKINWSKAPFYAYYKDFDIEGCAIPGPTTCSTNPKNWWEGVEYQALSAIEARRYRWVRMNHVIYDYCQDKSRYPMTPHECLSGI

>Medtr4g097640.1

MAIFYPFKNNGVFFMLLLWIVVSSVWGRPATFNQDFHVTWSEPHIKQIDQGRTIQLTLDQGSGCGFASKVKYLFGRVSMKIKLVPGDSAGTVTAFYMNSDTDSVRDELDFEFLGNRTGQPYTVQTNIYAHGKGDREQRVNLWFDPSADFHTYSILWNHHHIVFYVDEVPIRVYKNNEAKGIPYPKMQAMGVFSTLWEADNWATRGGLEKINWSKAPFYAYYKDFDIEGCAIPGPTTCSTNPKNWWEGVEYQALSAIEARRYRWVRMNHVIYDYCQDKSRYPMTPHECLSGI

>Medtr4g097700.1

MIGQTEDDKNKRGQAEDKTNCLDPTLISIPQSNIFFIKHIHIIHNIIQSMAIFSPFKHNSVFFILPATFNQDFHVTWSEPHIKQIDQGRTIQLTLDQGSGCGFASKVKYLFGRVSMKIKLVPGDSAGTVTAFYMNSDTDSVRDELDFEFLGNRTGQPYTVQTNIYAHGKGDREQRVNLWFDPSADFHTYSILWNHHHIVFYVDEVPIRVYKNNEAKGIPYPKMQAMGVFSTLWEADNWATRGGLEKINWSKAPFYAYYKDFDIEGCAIPGPTTCSTNPKNWWEGVEYQALSAIEARRYRWVRMNHVIYDYCQDKSRYPMTPHECLSGI

>Medtr2g049030.1

MYLSTLSFFLLHLIIAPLNIKGVNLDNVPFEKNYAPLWGKENIRILDQSQEVQITLNQNSGSGFQSLRKFGSGWFKLRTKLPQKDSSAVISTFYLKADAGDEIDFEFLGGNNKERPHILHTNIFTNGKGGREQRIRLWFDPAADFHNYTLLWNEKQLVFFVDDTPIRVFKNTTNKGGMYPTQAMKIIASIWNDTWASNGVPVNWNDAPFEAHYRGFGIDACQAQGTNTQECRSPKYWWNGEKFWGLNPRKIQAYKNIRSKYLIYDYCAKQPQIPECQNLPIY

>Medtr2g049130.1

MCLSPLSFLIVFLLGPLNVKGVDWDNVPFMQNYAPLWGQENMQILNQSREVQLTLNQESGSGFQSIHKYSSGWFNMKIKLPQNDSTEVITTFYLISVDGPTRDEIDFEFLGGNKERSHILHTNIFTNGQGGREQQFQFWFDPTADFHDYTFLWNEKQLVFFVDNIPIRVFKNNTDKGGSYPTQAMKIYATIWSSPWGSGGVPINWNEAPFEAHYRGFGINACQSQNPNIEQCNSFRYWWNTENYWELNFDQKQAYNNVRSKYLIYDYCTKQPENLECQGLHLN

>Medtr7g056090.1

MVGMLKQQSFFLFVTLFVAALICVSKAVEVSFKQNYKVTWGRNHVLFFDNGREVQLSFDKISGAGFRSKLEYSSGFFRMRIKIPTKDSLGVVTAFYVRNLSYRKHDEIDFEFLGNKGGPYTLQTNVFASDEGGREQRHSLWFDPTIDFHTYGILWNQHQIVFYVDETPIRIFKNKSNKGVSFPSNQMHVTVSIWNGEPWASNGKKIDWKQAPFLAQFQMFNIHGCQSHNPRKYDCYSPHLWWNGMKYWELNPQQQREYEDVRRIHLLYDYCSDRGQLHKECQIR

>Medtr8g013055.1

MSSLIYIFLVVVLVPHVVLAREIMRSEEIIFDQNYKVTWGDNHVISINQRKEIQLTMDYSSGSGFASKLTYGSGLFHMRIKVPGRDSAGVVTAYYLMSQGDSHDELDFEFLGNREGKPYALQTNVWANGEGDREQKIHLWFDPTTDFHDYKILWNPHQIVFYVDNIPIRVYKDNSNIGVGYPSKAMQVQASLWNGENWATDGGKAKINWTNAPFKANFQGFDVSGCQSQTLIDPNCASNNYWWNEQKFWQLDPAGQRQYENVKQNYVTYDYCKDRHRFPTPPLECLY

>Medtr3g089540.1

MASSSYYGSLFLLLLSLFLWFSTNVLGGNFNTLFDNLFGEERVDIKEDGNSMTLTLDEYCGSGIVSKNEYLFGRFDMKIKLVPGNSAGTVTAYYLSSVGAQHDEIDIEFLGNLTGEPYLLSTNVYAEGIGGREMQFYLWFDPTEDYHMYSIDWNPERIIILVDNNPIRVMLNRQRIGVPFPTKRPMRVYTTLWNGDSWATRWGEVKIDLTNAPFVAGFKNFNAIACIANQGQTANCRNYNGGKYKGLDRESKRKMKQVLSKWVVYDYCADLRRYAHGLPYECRKENRIQLD

>Medtr5g091730.1

MASYANNYVFLLLCLGLSFCTIAFGGNFNTDFNYLFGDFRANIQTGGNVASLQMDKYSGSGFGSKNAYLFGRFDMQIKLVPGNSAGIVTAYYLSSEGDHHDEIDIEFLGNVTGQPYILQTNIYANGVGGREMQYYLWFDPTQNFHTYSIDWNPQRIMILVDNQPIRVSRNKQGSGVPFPTNQPMRLYTTLWNGEAWATQGGTMKVDWSKGPFTAWFSNFNANACVPSQSNNCVGFNGGTNRGLSIDSRKKLNQIYSKWLVYDYCHDVRRYANGLPNECRRKSPRRMALED

>Medtr1g063020.1

MAFPFIFLFASLFFMASSSNAYWPPSPGYWPSSKVRSMSFYNGYRNLWGPQHQSMDQHGTTIWLDRTSGSGFKSNRPFRSGYFGASIKLHPGYTAGVITAFYLSNNEAHPGFHDEVDIEFLGTTFGKPYTLQTNVYIRGSGDGKIIGREMKFHLWFDPTKKFHHYAIVWNPKELIFLVDDMPIRRYPRKSDATFPLRPMWVYGSIWDASSWATEDGKYKADYKYQPFVAKYTNFKASGCTAYASRWCRPATASPYRSGGLTRQQHWSMSWVQRHHMVYNYCQDPKRDHRLTPECWG

>Medtr7g102260.1

MPLFVVAILFLMTFSSNAYWPPSPGYWPSSKFKSMNFYKGFTNRWGPQHQRLEQNALTIWLDRTSGSGFKSVRPFRSGYFGASIKLQPGYTAGVITSFYLSNNEAHPGYHDEVDIEFLGTTFGKPYTLQTNVYVRGSGDGKIVGREMKFHLWFDPTKGFHHYAILWSPKEIIFLVDDVPIRRYPRKSDTTFPIRPMWLYGSIWDASSWATEDGKYKADYRYQPFVAKYTNFKASGCTAYAPRWCHPVSASPYGSGGLNSQQNRAMSWVQRYHMVYNYCKDPKRDHRLTPECSG

>Medtr2g095800.1

MSRSPTLPLIFLPSSLCMAPLFSLFFSFLMCSRNAGGAPSPGYYPSSKVSTISFDQEFRNLWGAQHQKLDHGSLSIWLDSTTGSGFKSYHSYKSGYFGTAVKLQPGYTAGVNTNLYLSNIEDHKGTHDEIDIEFLGTTQDKPYVLQTNVYIRGSGDGKNIGREMRFHLWFDPTQDFHNYAILWKPSEIIFLVDDVPIRRYPRKSDATFPSRPMYVYGSIWDASAWATENGKYKANYTYQPFIGRYQNFKLQGCTTDSTSSCRPPSVSPPGYGGKLSRQQNVAMQWVQKNYLVYDYCHDPKRDHTLTPEC

>Medtr4g057450.1

MPLSSQSFNFFHMVPIFTFVVLSLTIFGSNAQGPPSPGYYPSSKISPISFSQGFRNLWGPQHQRLDQSSLTIWLDSNSGSGFKSLHSYKSGYFGAAIKLHPGYTAGVITSLYLSNNQDHPGNHDEIDIEFLGTTPGKPYVLQTNVYMRGSGDGNIIGREMQFHLWFDPTQDFHNYAILWKPSEIIFLVDDVPIRRYPRKSDATYPTRPMYLYGSIWDASSWATEDGKYKADYKYQPFVGRYTNFKLQGCTIQSPASCQSPSVSPSGYGSLSPQQYMAMQWVQSKYMVYNYCHDPRRNHNLIPEC

>Medtr8g064180.1

MTHLHQNLPFSFLLIFSLTTLVSSHSRPYSPPSVTHITNSFPHVTIESAFSNAFGASNVKFLSNGSMATLALDKISGSGLVSQSRYSYGFFSAAIKLPAGLSPGVVVAFYLSNADKFPHNHDEIDIELLGHDKRNDWVIQTNIYANGSVRTGREEKFYLWFDPTQQHHYYSILWNSYHTVFFVDNIPVREFIHKNTPSFIYPLKPMSLYATIWDGSEWATHGGKYPVNYKYGPFVVSLAEMELSGCINDPKSPISSCSKSNPSGLDPVDGAEFTKLSQQQIGAMDWARRKLMFYSYCNDRTRYKVLPPECQ

>Medtr8g090100.1

MEPDFLRNFSFAAFDLATIPFHDGFTYLWGKENVIPSLDGNTVKLIIHEHSGSGFKSIDMYTSGFFSAEIKLPSNYFATAGVCVTFYTTSIDVFDRRRHDELDFEFLGNKAGKPWQFQTNLFGNGTSGREERYDLWFDPTKEYHRYSILWTEINIIFFVDEIPIREVLRNKQMENNYPSKPMYLYATIWDASKWAIHEGNRKVDYSYGPFIATYKNLILKGCIFDPIEVPDDEFCSENLKAQDYAIMNPLRRLKMQNFRQRFMSYSYCYHTYRYPVPPPECVIVSKEKQMFNENGMLKVGRSRRRHSKRRGRASTTMGHMMF

>Medtr1g033420.1

MEKIQMMLFLFCFFIFVMPCYSVSPRNFPIIPFDEGYTPLFGDHNLIIHSDSKAVHLLLDESSGSGFASHDLYLHGYFSASIKLPSDYTAGVVVAFYLSNGDMYEKNHDEIDFEFLGNIRGKDWRIQTNVYGNGSTNIGREERYGLWFDPSEEFHHYSILWTDSKIIFYVDDVPIREVKRTESMRGDFPSKAMTLYSTIWDASTWATNGGKYKVNYKYAPYIAEFSDFVLHGCAVDPIEHVTKCDSAQSSETVPSDVTQLERIKMENFRLKHMTYSYCYDKTRYKVPLPECVIDSRQAERLRKFDPVTFGNGRHRRGKRHHQNKVTSF

>Medtr5g029100.2

MVILISLEMVTLFVPPMATVFNSSSIVTPVIILLNHIIHMQSSGFISSNMYQHGFFSANIKLPSNYSAGICVAFYTSNGDVFEKTHDELDFEFLGNIAGKPWRFQTNLYGNGSTHRGREERYRLWFDPTKGYHRYSILWTAKNVIFYIDEVPIREVLRNEEMGSEYPVKPMSLYATIWDASNWATSGGKYKVNYKYAPFVVEFKDLVLKGCSVDPIQEVSDNDVCSDQNVDLEAQDYANVTPMRRLAMRRFRQRFMYYSYCYDTIRYPVPPPECNIIPAEKQRFKETGRLKFGGSHRRHSRRGGRTSTPVEDTDQGDM

>Medtr5g029100.1

MNHHTLRCCGNIPKTHFLFSFFSLFFLFSFTNSAFDLATIPFNDGYSHLFGDGNVVRSSDGNGVQLLLDRYTGSGFISSNMYQHGFFSANIKLPSNYSAGICVAFYTSNGDVFEKTHDELDFEFLGNIAGKPWRFQTNLYGNGSTHRGREERYRLWFDPTKGYHRYSILWTAKNVIFYIDEVPIREVLRNEEMGSEYPVKPMSLYATIWDASNWATSGGKYKVNYKYAPFVVEFKDLVLKGCSVDPIQEVSDNDVCSDQNVDLEAQDYANVTPMRRLAMRRFRQRFMYYSYCYDTIRYPVPPPECNIIPAEKQRFKETGRLKFGGSHRRHSRRGGRTSTPVEDTDQGDM

>NtXTH1

MNNFSTLIFFVTAFIYLFHITLASIVSTGDFNKDFIVPWSPNHVNTSADGHTRSLIFDKESGSGIASNDTYLFGQFDMKIKLIPGNSAGTVVAFYLTSYQPNRDEVDFEFLGNVPGKPYTLQTNVYVDGLDDREQRINLWFDPTQDFHTYSILWNLHQIVFMVDRVPIRTYRNHADKGAKYPRWQPMALQISIWNGESWATDGGKTKIDWSKAPFVASLGNYTIDACVWKGNARFCRGESENNWWNKEKFSTLTWTQRRLFKWVRKYHLTYDYCMDNQRFQNNLPIECSLPKY

>NtXTH2

MKLKLVGGDSAGVVTAYYMCTEDGAGPTRDEVDFEFLGNRTGEPYLIQTNVYKNGTGGREMRHVLWFDPTEDFHSYSLLWNSHQLVFFVDEVPIRVYKNANYTNNFFPNEKPMYLFSSIWNADDWATRGGLEKTDWKNAPFVSTYKDFSVDGCQWEDPFPTCVSTTTKNWWDQYNSWHLSSDQKLNYAWVQRNLVIYDYCQDTKRYPEKPEECWLSPWD

>NtXTH3

MANLLLIAVLIAIYCSLSQAEVKGSFDDNFSKSCPESHFKTSEDGQIWYLSLDHKAGCGFMTRQKYRFGWFSMKLKLVGGDSAGVVTAYYMCTEDGAGPTRDEVDFEFLGNRTGEPYLIQTNVYKNGTGGREMRHVLWFDPTEDFHSYSLLWNSHQLVFFVDEVPIRVYKNTNYTNNFFPNEKPMYLFSSIWNADDWATRGGLEKTDWKNAPFVSTYKDFSVDGCQWEDPFPSCVSTTTENWWDQYNSWHLSSDQKLDYAWVQRNLVIYDYCQDTERYPEKPEECWLSPWD

>NtXTH4

MERMSSSIPKFLLIIALITVLFTLTQAEVQGSFDDNFSKSCPETHFKTSEDGQIWYLSLDKKAGCGFMTRQKYRFGWFSMKLKLVGGDSAGVVTAYYMCTEDGAGPTRDELDFEFLGNRTGEPYTIQTNVYKNGTGNREMRHILWFDPTEDFHTYSILWNTHQIVFFVDRVPIRVYKNANYTNNFFPNEKPMYLFSSIWNADDWATRGGLEKTNWKNQPFVSSYKDFSVDGCQWKDPFPACVSTTTKNWWDQYNSWHLSSDQKMDYAWVQRNLVTYDYCQDTERFPKKPEECWLNPWD

>NtXTH5

MEKMASSIPKILLIIALITVLFSLTQAEVQGSFDDNFSKSCPETHFKTSEDGQIWYLSLDKKAGCGFMTKQKYRFGWFSMKLKLVGGDSAGVVTAYYMCTEDGAGPTRDELDFEFLGNRTGEPYTIQTNVYKNGTGNREMRHILWFDPTEDFHTYSILWNTHQIVFFVDRVPIRVYKNANYTNNFFPNEKPMYLFSSIWNADDWATRGGLEKTNWKNQPFVSSYKDFSVDGCQWKDPFPACVSTTTKNWWDQYNSWHLSSDQKMDYAWVQRNLVTYDYCQDTERFPKKPEECWLNPWE

>NtXTH6

MERNASSMADLFFTAALMAALFSSSHAELIKGAFENNFSKSCPGTHFKTSQDGQIWYLTLDQISDCGFITKQSYRFGWFSTKLKLVGGDSAGVVTAFYMCSEVEAGPLRDEIDFEFLGNRTGQPYLIQTNVYNNGSGGREMRHLLWFDPTQDFHTYSILWNSHQIVFFVDKVPIRVYKNANHTNNFFPAERPMYVFSSIWNADNWATRGGLDKINWTSAPFIASYKDFILDACQWKDPFPACVSTTTQHWWDQYNAWHLSSKQKIDYAWVQRNFVVYDYCQDSVRNRYKPQECWLSALD

>NtXTH7

MERNMGDLLLFAALVATLFSSSHAQLIKGAFENTFSKSCPGTHFKTSQDGQIWYLTLDQVSDCGFITKQSYRFGWFSTKLKLVGGDSAGVVTAFYMCSEVEAGPLRDEIDFEFLGNRTGQPYLIQTNVYNNGSGGREMRHLLWFDPTQDFHTYSILWNSHQIVFFVDKVPIRVYKNANHTNNFFPAERPMYVFSSIWNADNWATRGGLDKINWTSAPFVASYKEFTLDACQWKDPFPACVSTTTQHWWDQYNAWHLSSKQKIDYTWVQRNFVVYDYCQDSVRNRYKPQECWLSPLD

>NtXTH8

MKQVIEYRCLLILGCGFASKSKYLFGRVSMKIKLVPGDSAGTVTAFYMNSDTDNVRDELDFEFLGNRSGQPYTVQTNVYVHGKGDKEQRINLWFDPSADFHTYTILWNHHHTVFYVDAVPIRVYKNNEAKGIPFPKFQPMGVYSTLWEADDWATRGGLEKINWSKSPFYAYYKDFDIEGCAMPGPANCASNPRNWWEGANYQQLSAVEARQYRWVRTNHMIYDYCTDKSRNPVPPPECVAGI

>NtXTH9

MISSSLKYSTVIPILLYALTFSSSVSARPATFLQDFKVAWADSHIKQIDGGKAIQLILDQNSGCGFASKSKYLFGRVSMKIKLVPGDSAGTVTAFYMNSDTDNVRDELDFEFLGNRSGQPYTVQTNVYVHGKGDKEQRINLWFDPSADFHTYTILWNHHHTVFYVDAVPIRVYKNNEAKGIPFPKFQPMGVYSTLWEADDWATRGGLEKINWSRSPFYAYYKDFDIEGCAMPGPANCASNPRNWWEGANYQQLSAVEAKQYRWVRMNHMIYDYCTDKSRNPVTPPECVAGI

>NtXTH10

MGKLTSLKYSAAILILLYALTFSFSVSARPATFLQDFKVSWAYSHIKQIDGGRAIQLILDQNSGCGFASKSKYLFGRVSMKIKLVPGDSAGTVTAFYMNSDTDNVRDELDFEFLGNRSGQPYTVQTNVYVHGKGDKEQRVNLWFDPSADFHTYTILWNHHHAVFYVDAVPIRVYKNNEAKGIPFPKFQPMGVYSTLWEADDWATRGGLEKINWSKSPFYAYYKDFDIEGCAMPGPANCASNPRNWWEGANYQQLSAVEARQYRWVRMNHMIYDYCTDKSRNPVTPPECVAGI

>NtXTH11

MARLTSLKYSAAILILLYALTFSFSVSARPATFLQDFKVSWSDSHIKQIDGGRAIQLILDQNSGCGFASKSKYLFGRVSMKIKLVPGDSAGTVTAFYMNSDTDNVRDELDFEFLGNRSGQPYTVQTNVYVHGKGDKEQRVNLWFDPSADFHTYTILWNHHHAVFYVDAVPIRVYKNNEAKGIPFPKFQPMGVYSTLWEADDWATRGGLEKINWSKSPFYAYYKDFDIEGCAMPGPANCASNPRNWWEGANYQQLSAAEARQYRWVRMNHMIYDYCTDKSRNPVTPPECVAGI

>NtXTH12

MVSFPMEFKWVFLGISLMLVGLVSSSRFEELYQPSWATDHLTNEGEILRMKLDNLSGAGFSSKNKYMFGKVTVQIKLVEGDSAGTVTAFYMSSEGPTHNEFDFEFLGNTTGEPYSVQTNVYVNGVGNREQRLNLWFDPSNEFHSYSILWNQHRVVFLVDETPVRVHSNLEHKGIPFPKDQAMGVYSSIWNADDWATQGGRVKTDWSHAPFIASYRGFEIDGCECPATVAAAENSKRCSSSAEKRYWWDEPTMSELSLHQSHQLIWVRANHMVYDYCTDTARFPVAPVECQHHQHKTRN

>NtXTH13

MVSFPMEFKCVFLGISLIMVGLVSSSRFEELYQPSWATDHLTNEGEILRMKLDNLSGAGFSSKNKYMFGKVTVQIKLVEGDSAGTVTAFYMSSEGPTHNEFDFEFLGNTTGEPYSVQTNVYVNGVGNREQRLNLWFDPSKEFHSYSILWNQRRVVFLVDDTPIRVHSNLEHKGIPFPKDQAMGVYSSIWNADDWATQGGRVKTDWSHAPFIASYRGFEIDGCECPATVAAAENSKRCSSSAVKRYWWDEPVMSELSLHQSHQLIWVRANHMVYDYCTDTARFPVAPVECQHHQHKFHN

>NtXTH14

MPSSMIVFLILAMLLNTGVGVNFAEVFESSWAPDHITVVGDQVMLTLDNASGCGFQSKNKYLFGKASVQIKLVGGDSAGTVIAFYMSSEGANHDELDFEFLGNVSGEPYLVQTNVYANGTGDREQRHSLWFDPTTDFHTYSFFWNHHTIIFSVDDIPIRVFQNKENKGVAYPKNQGMGIYGSLWNADDWATQGGRVKTNWSHSPFVATFRAFEIDACDLSGEDTVAAGAKCGKLAECWWDKPAVKQLNKSKKRQFKMVQSKHLVYDYCKDTARFTQMPKECLD

>NtXTH15

MRRKSCMLTTVPWLPLKHSLARWVGVNFTEVFESSWSPDHITVVGDQVMLTLDNASGCGFQSKNKYMFGKASAQIKLVDGDSAGTVIAFYMSSEGANHDELDFEFLGNVSGEPYLVQTNVYANGTGDREQRHSLWFDPTADFHTYSFFWNHHTIIFSVDDIPIRVFKNTEKKGVAYPKNQGMGVYGSLWNADDWATQGGRVKTNWSHSPFVATFRAFEIDACDLSGEDTVAAGAKCGKLAQCWWDKPAMRELNKSKKRQFKMVQSKHLVYDYCKDTARFTQMPKECLD

>NtXTH16

MGMNMLLVCVLFVVGAMAAAPKKPMDVPFGRNYENTWAPDHVKYFNGGSEIQLFLDNRTGTGFQSKGSYLFGHFAMHIKMVAGDSAGTVTAFYLSSQNNEHDEIDFEFLGNKTGEPYVVQTNIYTGGKGDKEQRIYLWFDPTKDYHTYSVLWNLHQIVFFVDEYPIRTFKNSKDLGVKFPFDQPMKIYSSLWEADDWATRGGLEKIDWSNAPFVASYKGFHIDGCEASVNAKLCANQGKKWWDQKEFQDLDKQQWRLLRRVRDKYTIYNYCTDKKRFATLPKECRRNRDVPRKSSKKSP

>NtXTH17

MGFKWMNMLLFCALFVVGAMAAAPKKPMDVPFGRNYENSWAPDHVKYFNGGSEIQLFLDNRTGTGFQSKGSYLFGHFAMHIKMVAGDSAGTVTAFYLSSQNNEHDEIDFEFLGNKTGEPYVVQTNVYTGGKGDKEQRIYLWFDPTKDYHTYSVLWNLHQIVFFVDEYPIRTFKNSKDLGVKFPFDQPMKIYSSLWEADDWATRGGLEKIDWSNAPFVASYKGFHIDGCEASVNAKYCSNQGKKWWDQKEFQDLDKQQWRLLRRVRDKYTIYNYCTDKKRFATMPKECRRNRDVPRKSSKKSP

>NtXTH18

MGLKGLLFSIVLINLSLLGLCGYPRKPVDVPFWKNYEPSWASHHIKYLNGGSTADLVLDRSSGAGFQSKKSYLFGHFSMKLRLVGGDSAGVVTAFYLSSNNAEHDEIDFEFLGNRTGQPYILQTNVFTGGKGDREQRIYLWFDPTKGYHSYSVLWNTFQIVIFVDDVPIRAFKNSKDLGVKFPFNQPMKIYSSLWDADDWATRGGLEKTDWSNAPFTASYTSFHVDGCEAATPQEVQVCNTKGMRWWDQKAFQDLDALQYRRLRWVRQKYTIYNYCTDRKRYPTLPPECTKDRDI

>NtXTH19

MGVKGLLFSIVLINLSLLGLCGYPRKPVDVPFWKNYEPSWASHHIKYLSGGSTVDLVLDRSSGAGFQSKKSYLFGHFSMKLKLVGGDSAGVVTAFYLSSNNAEHDEIDFEFLGNRTGQPYILQTNVFTGGKGDREQRIYLWFDPTKGYHSYSVLWNTFQIVIFVDDVPIRAFKNSKDLGVKFPFNQPMKIYSSLWDADDWATRGGLEKTDWSNAPFTASYTSFHVDGCEAATPQEVQVCNTKGMRWWDQKAFQDLDALQYRRLRWVRQKYTIYNYCTDRKRYPTLPPECTKDRDI

>NtXTH20

MQLKLVPGNSAGTVTTFFLSSQGAGHDEIDFEFLGNVSGQPYTVHTNVYSQGKGNKEQQFHLWFDPTAAFHTYSIIWNAQKIIFLVDNSPIRVYNNHESAGIPFPKSQPMKVYCSLWNADEWATQGGRVKTDWTHAPFTAYYRNFNIDGCAVTSGASSCKSTDSANNARPWQNQELDAKGRNRLRWVQSRHMVYNYCADSKRFPQGFSHECKRSRFL

>NtXTH21

MSPRFSFKMLILPIVMASLWAAASAGNFYNLADITWGEGRGKITEGGRGLSLSLDKLSGSGFQSKNEYLFGRFDMQLKLVPGNSAGTVTTFFLSSQGAGHDEIDFEFLGNVSGQPYTVHTNVYSQGKGNKEQQFHLWFDPTAAFHTYSIIWNAQKIIFLVDNSPIRVYNNHESNGIPFPKIQPMKVYCSLWNADEWATQGGRVKTDWTHVPFTAYYRNFNIDGCAVTSGTSSCKSTDSANNARPWQNQELDAKGRNRLRWVQSRHMVYNYCADSKRFPQGFSHECKRSRFL

>NtXTH22

MASHLFLISILMGSLVAASANFNNLAEITWGEGRGKITEGGKGLSLSLDKLSGSGFQSKNEYLFGRFDMQLKLVPGNSAGTVTTFFLSSQGEGHDEIDFEFLGNTTGEPYTVHTNVYSQGKGNKEQQFHLWFDPTAAFHTYTIVWNSNRIVFLVDNIPIRVYNNHENNGIPFPKSQPMKVYCSLWNADEWATQGGRVKTDWTHAPFTAYYRNFKIDGCAVTSGASSCKSTDSAGNAKAWQNQELDAKGRNRVRWVQSRHMVYNYCADKKRFPQGYSHECKSSRF

>NtXTH23

MASHFLLISILMGSLVVASANFNNLAEITWGEGRGKITEGGKGLSLSLDKLSGSGFQSKNEYLFGRFDMQLKLVPGNSAGTVTTFFLSSQGKGHDEIDFEFLGNTTGEPYTVHTNVYSQGKGNKEQQFHLWFDPTAAFHTYTIVWNANRILFLVDNIPIRVYNNHESNGIPFPKSQPMKVYCSLWNADEWATQGGRVKTDWTHAPFTAYYRNFKIDGCAVTSGASSCKSTDSAGNAKAWQNHELDAKGRNRVRWVQSRHMVYNYCADKKRFPQGYSHECKSSRF

>NtXTH24

MASKFSSVMLLLCIIMSIQLLAASAGNFYRDAVITWGEGRGKIQEGGRGLALTLDKLSGSGFQSKNEYLFGRFDMQLKLVPGNSAGTVTTFFLSSQGEGHDEIDFEFLGNVSGQPYTVHTNVYTQGKGNKEQQFHLWFDPTAAFHTYTIVWNPHRIVFLVDNSPIRVYNNHESIGIPFPKSQAMRVYCSLWNADEWATQGGRVKTDWTLAPFTAYYRNINIDGCAVLSGTSSCKSSNSANNAKPWQTHELDGKGRNRLRWVQSRHMVYNYCADSKRFPQGFSAECKSSRF

>NtXTH25

MASKFSSAMLLLCILMSIQLLAASAGNFYRDTVITWGEGRGKIQEGGRGLALTLDKLSGSGFQSKNEYLFGRFDMQLKLVPGNSAGTVTTFFLSSQGEGHDEIDFEFLGNVSGQPYTVHTNVYTQGKGNKEQQFHLWFDPTAAFHTYTIVWNPHRIVFLVDNSPIRVYNNHENIGIPFPKSQAMRVYCSLWNADEWATQGGRVKTDWTLAPFTAYYRNINIDGCAVLSGTSSCKSSNSANNAKPWQTHELDGKGRNRLRWVQSRHMVYNYCADSKRFPQGFSEECKRSRF

>NtXTH26

MSLSSASSRIPKMFLQLSVLAVFLLCTACADNFYQDATVTWGDQRAHIQEGGRLLTLSLDKISGSGFQSKSEFLFGRFDMQLKLIPGNSAGTVTTFYLSSQGAGHDEIDFEFLGNSSGQPYTVHTNVYSQGKGNKEQQFHLWFDPTTSFHTYSIIWNAQRIIFLVDNIPIRVYNNHEALGVAFPKNQAMRVYASLWNADDWATQGGRVKTDWSMAPFTASYRNFNTNACVWSAASSTSSCGGSKSTDSANNDQTWQTQELDANGRNRLRWVQQKYMTYNYCTDAQRFNQVIPPECKRSRF

>NtXTH27

MGSRIFLVLALVFSSCMVSYGGNFFQEFDFTWGGNRAKIFNGGQLMSLSLDKVSGSGFQSKKEYLFGRIDMQIKLVAGNSAGTVTTYYLSSQGPTHDEIDFEFLGNVTGEPYILHTNIYAQGKGNKEQQFYLWFDPTKNFHTYSIIWKPQHIIFLVDNTPIRVYKNAESIGVPFPKNQPMRIYSSLWNADDWATRGGLVKTDWSKAPFTAYYRNFNSQTFSSSQFSNEKWQNQELDANGRRRLRWVQRNFMIYNYCTDFKRFPQGFPPECKRF

>NtXTH28

MARFSSSSSRSRSSLPYIVLLFVAALFVFKIDVIISQTFSSARRNLENTPNRILVKSKSQETDDSIPVVLVNGTFHRHFILSWGDDRGKIHENGELLTLSLDKQSGSGFQSKKEYLFAKIDMQIKLVPGNSAGTVTTFYLSSQGNKHDEIDFEFLGNSTGNPYTLHTNIFSLGQGNREQQFFLWFDPTADYHTYSILWNPKCIIFYVDGTPIREFKNAEKIGVPFLKYQPMRLYSSLWNADDWATQGGRVKTNWKLAPFIASYKNFTYEACIYSRLTSSSSCNINSPPFGNNAWLTHELDRRSRAKMKILQKKHMIYDYCKDKWRFPKGPAPECKLQ

>NtXTH29

MARFSSSSSRSRSSLPYIILLFVAALFVFKIDVIISQSFSSARRNLENTPNHILVKSKSQETDDSIPVVLVNGTFHRHFILSWGDDRGKIHENGELLTLSLDKLSGSGFQSKKEYLFAKIDMQIKLVPGNSAGTVTTFYLSSQGNKHDEIDFEFLGNSTGNPYTLHTNIFSLGQGNREQQFFLWFDPTADYHTYSILWNPKCIIFYVDGTPIREYKNAEKIGVPFPKYQPMRLYSSLWNADDWATQGGRIKTNWKLAPFIASYKNFTYDACIYSRLTSSSSCNINSPPFGNDSWLTHELDRRSRAKMKILQKKHMIYDYCNDKWRFPKGPAPECKLQ

>NtXTH30

MMKTSSCMFSFLFLSFLVLVALAENFNQEFDVTWGDGRVKILENGQLLTLSLDKTSGSGFRSKRQYMFGKIDMKIKLVPGNSAGTVTTYYLSSLGPTHDEIDFEFLGNLSGDPYILHTNVFVQGKGEREQQFYLWFDPTKDFHTYSILWNPRSIIFSVDGTPIRQFKNLEASRGIPYPKNQPMWIYSSLWDAEDWATRGGLVKTDWSKAPFIASYRNFNAQACVWSSGSTSSCSINSTANSWITESLDNSGQARIKWVQKNYMVYNYCTDTKRFPQGFPLECSLN

>NtXTH31

MMKTSISCIISFLFLSFLLVVMAALAGDFNQEFDVTWGDGRVKILENGQLLTLSLDKTSGSGFRSKRQYMFGKIDMKIKLVPGNSAGTVTTYYLSSLGPTHDEIDFEFLGNLSGDPYILHTNVFTQGKGDREQQFYLWFDPTKDFHTYSILWNPRSIIFSVDGTPIRQFKNLETSMGIPYPKNQPMWIYSSLWDAEDWATRGGLVKTDWSQAPFVASYRNFNAQACVWSSGSTSSCSRNSTANSWITESLDNSGQARIKWVQKNYMVYNYCTDIKRFPQGFPLECSLN

>NtXTH32

MMKSFLFQMMFLVVAFAGNFNQNFDITWGDGRAKILENGQLLTLSLDKTSGSGFRSKNQYLFGKIDLKIKLVPGNSAGTVTTYYLSSIGSSHDEIDFEFLGNLSGDPYILHTNVFTQGKGNREQQFYLWFDPTKYFHTYSILWNPQSIIFSVDGTPIRQFKNLEASGIPYPKNQPMWIYSSLWNADDWATRGGLVKTDWSKAPFIASYRNYNAQACVWSSTSSSSCSPNNSTENSWLSESLDNTGQSKIKWVQNNYMIYNYCTDTKRFPQGFPPECSLN

>NtXTH33

MMKSFLFLMIFLVVALAGNFNKDFDITWGDGRAKILENGQLLTLSLDKTSGSGFRSKNQYLFGKIDLKIKLVPGNSAGTVTTYYLSSIGSSHDEIDFEFLGNLSGDPYILHTNVFTQGKGNREQQFYLWFDPTKDFHTYTILWNPQSIIFSVDGTPIRQFKNLEASGIPYPKNQPMWIYSSLWNADDWATRGGLVKTDWSKAPFIASYRNYNAQACVWSSSSSSSCTSNSSTGNSWLSESLDSTGQSRIKWVQSNYMIYNYCTDTKRFPQGFPPECSLN

>NtXTH34

MSSFSSKLVLALIVSAFAIAIAGTIDENFEITWGEGRAKMLNNGELLTLSLDKISGSGFQSKNEYLFGKIDMQLKLVPGNSAGTVTAYYLSSQGPTHDEIDFEFLGNLSGDPYTLHTNVFSQGKGNREQQFHLWFDPTADFHTYSILWNPQRIIFYVDGTPIREYKNAESIGVSYPKKQPMRIYSSLWNADDWATRGGLIKTDWSKAPFSASYRNFKSATSTSAATSNSWLNEELDNTSQERLKWVQKNYMVYNYCNDSKRFPQGFPADCAM

>NtXTH35

MASLLAQYLVFLALCSLQYHSLAYNNFNQDFDVTWGDGRAKVLNNGKLLTLSLDKASGSGIQSKREYLFGRIDMQLKLVRGNSAGTVTTYYLSSQGATHDEIDFEFLGNLSGDPYIIHTNVYTQGKGDKEQQFYLWFDPTAGFHTYSILWNPQTIIFYVDGTPIRVFKNMKSRGIPYPNKQPMRVYASLWNADDWATRGGLIKTDWSNAPFIASFRNFKANACVWEFGKSSCNSSTNPWFFQELDSTSQAKLQWVQKNYMVYNYCTDIKRFPQGFPLECNFNSTTS

>NtXTH36

MASLLVQCLNFLALCSLQYHILASSNFNQDFDVTWGDGRAKVLNNGKLLTLSLDKASGSGIQSKREYLFGRIDMQLKLVRENSAGTVTTYYLSSQGATHDEIDFEFLGNLSGDPYIIHTNVYTQGKGDKEQQFYLWFDPTAGFHTYSILWNPQTIIFYVDGTPIRVFKNMKSSGVPYPTNQPMRVYASLWNADDWATRGGLIKTDWSKAPFIASFRNFKANACVWEFGKSSCNSSTNSTKPWFFQELDSTSQARLQWVQKNYMVYNYCTDIKRFPQGLPQECNFNSTTS

>NtXTH37

MAKFIAFNSLVLIIATFAFHCAIVNAKISSSMYINWGAHHCQMLGDDLQLVLDKSAGSGAQSKRTFLFGSFEMLIKLVPNNSAGTVTTYYLSSTGTKHDEIGFEFLGNVSGQPYIIHTNIYTQGVGNKEQQFYPWFDPTADFHNYTIHWNPNAVVWYIDGIPIRVFRNYQLKGIPFPNQQGMRIYSSLWNADEWATRGGRDKIDWTNAPFIATYRKFRPRACYWNGPLSIVQCAIPTKSNWWNFPLYSKLSAPKVDQMNSIRSKYMIYDYCKDTTRFKGVMPTECTLPQN

>NtXTH38

MAKFIAFNSLVLIIATIAFHCAIVNGKISSSMYVNWGAHHCQMLGDDLQLVLDKSAGSGAQSKRTFLFGSFEMLIKLVPNNSAGTVTTYYLSSTGTKHDEIDFEFLGNVSGQPYILHTNIYTQGVGNREQQFYPWFDPTADFHNYTIHWNPNAVVWYVDGIPIRVFRNYQFKGIPYPNQQGMRIYSSLWNADEWATRGGRDKIDWTNAPFIATYRKFRPRACYWNGPLSIVQCAIPTKSNWWNSPLYSKLSAPKVDQMNSIRSKYMIYDYCKDTTRFKGVMPIECSLPQY

>NtXTH39

MAKFVAFNSLVLIIATIAFHCAIVNGKISSSMYVNWGAHHCQMLGEDLQLVLDKSAGSGAQSKRTFLFGSFEMLIKLVPNNSAGTVTTYYLSSTGTKHDEIDFEFLGNVSGQPYILHTNIYTQGVGNREQQFYPWFDPTADFHNYTIHWNPNAVVWYVDSIPIRVFRNYQLKGIPFPNQQGMRIYSSLWNADEWATRGGRDKIDWTNAPFIAKYRKFRPRACYWNGPLSIVQCAIPTKSNWWNSPLYSKLSAPKVDQMNSIRSKYMIYDYCKDTTRFKGVTPTECSLPQN

>NtXTH40

MAKFITFSLVLIIATFAFRCTLVNGKISSSMYINWGAHHCKMQGDDLQLVLDKSAGSGAQSKRTFLFGSFEMLIKLVPNNSAGTVTTYYLSSTGTKHDEIDFEFLGNVSGQPYIIHTNIYTQGVGNKEQQFYPWFDPTADFHNYTIHWNLNAVVWYVDGIPIRVFRNYELKGIPFPNQQGMRIYSSLWNADEWATRGGRDKIDWTNAPFIATYRNFRPRACYWNGPLSIGQCAIPTKSNWWNSPLYNKLSAPKVDQMNSIRSKYMIYDYCKDTKRFKGVTPTECSLPQN

>NtXTH41

MFKIMASSRLLSLANLFILAIAFHLVSVNGMFSDNMYIGWGAHHSWMQGNDLQLVLDQSSGSGVQSKGAFLFGSIQMQIKLVPGNSAGTVTAYYLSSTGDKHDEIDFEFLGNVSGHPYIIHTNIFTQGAGGREQQFYPWFDPTADYHNYTIHWNPSAVVWYVDDIPIRVYKNYQSQGILYPNAQGMGVYSSLWNADNWATRGGLDKIDWTNAPFIAKYRNFAPRACPWYGPGSISHCAAPTPNNWYTSPEYSQLSYAKQGQMNWVRNNYMIYDYCKDTTRFNGQIPGECFKPQF

>NtXTH42

MFKIMASSRLLSLSNLFILAIAFHLVSVNGMFSDNMYINWGAHHSWMQGNDLQLVLDQSAGSGVQSKGAFLFGSIEMQIKLVPGNSAGTVTAYYLSSTGDKHDEIDFEFLGNVSGQPYIIHTNIFTQGAGGREQQFYPWFDPTADYHNYTIHWNPSAVVWYVDGIPIRVYKNYQSQGILYPNAQGMKVYSSLWNADNWATRGGLDKIDWTNAPFIAKYRNFAPRACPWYGPGSIRQCAAPTPNNWYTSYEYSQLSYAKQGQMNWVRNNYMIYDYCKDKTRFNGQIPGECFKPQI

>NtXTH43

MAIFFLHFLLLLIVVPSTNAGYWPPSPGYYPSSKFRSMSFYQGFRNLWGPNHQNVDNNGINIWLDRNSGSGFKSIKPFRSGYFGASIKLQPGYTAGVITAFYLSNNEAHPGYHDEVDIEFLGTTFGKPYTLQTNVYIRGSGDGKIVGREMKFHLWFDPTKEFHHYAILWSPREIIFLVDDVPIRRYARKSIATFPLRPMWLYGSIWDASSWATEDGKYKADYRYQPFYGKFTNFKASGCTAYSSRWCHPVSASPSRSGGLTRQQRQAMNWVHSHYLAYDYCRDSKRDHSLTPECWR

>NtXTH44

MSIFFLPFLLFLIVLPSTNAGYWPPSPGYYPSSKFKSMSFYQGFKNLWGPNHQNVDNNGINIWLDRNSGSGFKSIKPFRSGYFGASIKLQPGYTAGVITAFYLSNNEAHPGYHDEVDIEFLGTTFGKPYTLQTNVYIRGSGDGKIIGREMKFHLWFDPTKDFHHYAILWSPREIIFLVDDVPIRRYARKSIATFPLRPMWLYGSIWDASSWATEDGKYKADYRYQPFYGKFTNFKASGCTAYSSRWCHPVSASPSRSGGLTRQQRQAMNWVHSHYLAYDYCRDSKRDHSLTPECWR

>NtXTH45

MANLFLLSLLLIFLFNSSNAQGPLSPGYYPSSKVQSLGFNQGFRNLWGPQHQSLDQSALTIWLDKTSGGSGFKSLENYRSGYFGTSVKLQPGYTAGIITSFYLSNNQDYPGNHDEIDIEFLGTTPNKPYTLQTNVYIRGSGDGNIIGREMKFHLWFDPTKAYHNYAILWDPNEIIFFVDDVPIRRYPRKNDATFPQRPMYVYGSIWDASSWATEEGRIKADYRYQPFVGKYNNFKIAGCTANENPWCGRSPSSSPSRAGGLSRQQIAAMLWVQRNYKVYDYCRDPRRDHTHTPEC

>NtXTH46

MALFLLSLLLLFLFNSSNAQGPPSPGYYPSSKVQSLGFSQCFRNLWGPQHQSLDQSALTIWLDKTTGGSGFKSLKNYRSGYFGTSVKLQPGYTAGIITSFYLSNNQDYPGNHDEIDIEFLGTTPNKPYTLQTNVYIRGSGDGNIIGREMKFHLWFDPTQAYHNYAILWNPNEIIFFVDDVPIRRYPRKNDATFPQRPMYVYGSIWDASSWATEEGRIKADYRYQPFIGKYNNFKIAGCTANENPWCGRSPSSSSSRAGGLSRQQMAAMLWVQRNYKVYDYCRDPRRDHTHTPEC

>NtXTH47

MDFFHHNKTFLLSQFLIFCMIVVVSCRGPVYKPPEVEKLTDHFSRLSVNQGYNVFFGGANVRMTNNGSSADLILDKSSGSGLISKEKYYYGFFNAALKLPAHFTSGVVIAFYMSNSDVFPHNHDEIDFELLGHDKRRDWVLQTNLYGNGSVHTGREEKFYLWFDPTLDFHDYTILWNNHHIVFLVDNVPIREVVHNTAISSVYPSKPMSVIATIWDGSEWATHGGKYPVNYQYAPFVTSMKEVELEGCVRQQNTSATSTCFRRSTSSLDPVDGEEFMKLSQQQMTGLDWVRRKHMFYSYCQDTNRYKVLPPECTSN

>NtXTH48

MEFYHQHKTCLFSGFLIFCMIAVASSLGPIYTPPEAERLTDRFSRLSVNQGYNVFFGGANVRLTNNGSNADLILDKSSGSGLVSRDKYYYGFFNAALKLPANFTSGVVVAFYLSNQNIFPHNHDELDFELLGYDKRRDWVLQTNIYGNGSVSTGREEKFYLWFDPTQDFHDYSILWNNHHILFLVDNVPVREVVNNTTISSVYPSKPMSIYATIWDGSQWATRGGKYPVNYTYAPFVTSIKGVELEGCVSEQNASAASACARRSTSSLDPVDGEEFVKLSQQQMTGLDWARRKHMFYSYCQDTRRYKVLPPECTAT

>NtXTH49

MEFFHQHNTLLLSEFLIFCMISVASSLGPIYTPPEVERLTDRFSRLSVNQGYNMFFGGVNVRLTNNGSSADLILDKSSGSGLVSRDKYYYGFFNAALKLPANFTSGVVVAFYLSNQNIFPHDHDELDFELLGYDKRRDWVLQTNNYGNGSVSTGREGKFYLWFDPTQDFHDYTILWNNHHILFLVDNVPVREVVHNTAISSVYPSKPMSIYVTIWDGSQWATRRGKYPVNYTYAPFVTSIKGVELEGCVSEQNGSAATACARRSTSSLDPVDGEEFVKLSQQQMMGLDWARRKHMFYSYCQDTRRYKVLPPECTAT

>NtXTH50

MDYRVLSSLSKSLTPFSLLMLLYIFPAAETATATTAKAFNLSTITFEEGYSPLFSDFNIERSPDDTSFRLLLNRFSGSGVISTEYYNYGFFSASIKLPAIYTAGIVVAFYTSNVDTFEKNHDELDIEFLGNVNGQPWRFQTNLYGNGSVSRGREERYRMWFDPSNDFHHYSILWTPKNIIFYVDETPIREVNRNPAMGGDFPSKPMSLYATIWDASSWATNGGKAKVDYKHEPFATEFKDLVLEGCIVDPIEQISSTNCTDRIARLLSQNYSIMTPERRKSMKWFRERYMYYSYCYDNIRYPVPPPECVIVQSERDLFKDSGRLRQKMKFGGSHSHRKHRPGRSSRRRNRAAGGGSSKSGQAAAM

>NtXTH51

MDFIRKKICLSVFLFFHVWFSTALNVSTIPFSDGFSHLFGEGNILHATDDKSLQLHLNQRTGSGFKSSDLYNHGFFSAKIKLPSDYTAGIVVAFYTTNGDLFTKTHDELDFEFLGNIRGKAWRFQTNMYGNGSTSRGREERYYLWFDPSKEFHRYSILWTNKNIIFYIDDVPIREIVRNDAMGGDYPSKPMGLYATIWDASDWATSGGKYKTNYKYAPFIAEFTDLVLNGCAMDPLEQVVNNPSCDEKDDELQKADFSRITPRQRMAMKRFRSKYMYYSYCYDSLRYSVPPPECEIDPIEQQHFKETGRLKFNKHHHRHPKRTKSQVLDARNYGNQDEE

>NtXTH52

MDFIRKKICLSVFLFFHVCFITADAALNVSTIPFSDGFSHLFGEGNILHATDDKSLQLHLNQRTGSGFKSSDLYTHGFFSAKIKLPSDYTAGIVVAFYTTNGDLFTKTHDELDFEFLGNIRGKAWRFQTNMYGNGSTSRGREERYYLWFDPSKEFHRYSILWTIKNIIFYIDDVPIREIVRNDAMGGDYPSKPMGLYATIWDASDWATSGGKYKTNYKYAPFIAEFTDLVLNGCAMDPLEQVVNNPSCDEKDDELQKADFSRITPRQRMAMKRFRSKYMYYSYCYDSLRYSVPPPECEIDHVEQQHFKETGRLKFNKHGHHRHAKRTRSQVLDARNHGNQDEE

>NtXTH53

MVNYHLVTFIFFSVVELVYGSSRNLPILAFDEGYSHLFGDDNVMILKDGKSAHISLDERTGAGFVSQDLYLHGFFSASIKLPADYTAGVVVAFYMSNVDMFEKNHDEIDFEFLGNIRGKDWRIQTNIYGNGSTSVGREERYGLWFDPSEDFHHYSILWTENFIIFYVDNVPIREIKRTEAMGGDFPSKPMSLYATIWDGSGWATNGGKYKVNYKYAPYIAKFSDFVLHGCAVDPIELSSKCDTAPKTASIPTGITPDQRRKMEKFRKKQMQYSYCYDKTRYKVPPPECVIDPKEAERLRAFDPVTFGGSRHHHGKQHRRSRSRAEGDISFL

>NtXTH54

MVNYHLVIFIFFSVVELVYGSSRNLPILAFDEGYSHLFGDNNLMILKDGKSAHISLDERTGAGFVSQDLYLHGFFSASIKLPADYTAGVVVAFYMSNVDMFEKNHDEIDFEFLGNIRGKDWRIQTNIYGNGSTSFGREERYGLWFDPSEDFHHYSILWTENFIIFYVDNVPIREIKRTEAMGGDFPSKPMSLYATIWDGSGWATNGGKYKVNYKYAPYIAKFSDFVLHGCAVDPIELSSKCDTAPKTSSIPTGITPDQRRKMENFRKKQMQYSYCYDKTRYKVPPTECVIDPKEAERLRVFDPVTFGGSRHHHGKRHSRSRSRAEGDVSFL

>NtXTH55

MVNFRLEIFILCSFLVLVCGSSKQLQTLPFDEGYSQLFGHDNLMVLEDGKSVHLSLDERTGAGFVSQDLYLHGYFSASIKLPADYTAGVVVAFYMSNGDMFEKNHDEIDFEFLGNIRAKKWRIQTNIYGNGSTNVGREERYGLWFDPSEDFHQYSILWTESQIIFYVDNIPIREIKRTKAMGGDFPSKPMSLYATIWDGSSWATNGGKYKVNYKYAPYVAKFSDFILHGCAVDPIELSPKCDTTPNSASIPTSISPDQRRKMESFRKKYLQYSYCYDRTRYNVPLSECVIDPKEADRLRGFDPVTFGGVQRHHSKRHHQRQSRREDTSSE

>NtXTH56

MVNFRLGIFILCSFLVLVSGSSKKLQTLPFDEGYSQLFGHDNLMVLEDGKSVHISLDERTGAGFVSQDLYLHGYFSASIKLPADYTAGVVVAFYMSNGDMFEKSHDEIDFEFLGNIRAKNWRIQTNIYGNGSTNVGREERYGLWFDPSEDFHQYTILWTESQIIFYVDNIPIREIKRTKAMGGDFPSKPMSLYATIWDGSSWATNGGKYKVNYKYAPYVAKFSDFVLHGCAVDPIELSPKCDTAPKSAFVPTGISPDQRRKMESFRKKYLQYSYCYDRTRYNVPLSECVIDPKEADRLQGFDPVTFGGVQRHHSKRRRQRQSRREDASSE
