# Supplementary material for: Transcriptome-wide characterization and functional analysis of Xyloglucan endo-transglycosylase/hydrolase (XTH) gene family of Salicornia europaea L. under salinity and drought stress
Source: BMC Plant Biol. 2021 Oct 25;21:491. doi: 10.1186/s12870-021-03269-y (PMC8547092; doi:10.1186/s12870-021-03269-y)
Supplement: Supplementary file 4 — Additional file 4: Supplementary file 4. Amino acid sequence of the predicted 35 SeXTH proteins. [file 12870_2021_3269_MOESM4_ESM.docx]

Supplementary file 4: Amino acid sequence of the predicted 35 SeXTH proteins.

>SeXTH1

SSSTTIFITLFVTTFIAFSTADFNKDFAITWGNGRAKILNNGDDLTLSLDKASGSGFQSKNEYLFGKIDMQLKLVPGNSAGTVTAYYLSSQGPTHDEIDFEFLGNVTGEPYTLHTNVFAQGKGSREKQFHLWFDPTKDFHTYSILWNPERIVFSVDGTPIREHKNMESKGIPFPKNQPMRIYSSLWEADDWATQGGRVKTDWTHAPFTASYRNFNADACVWASGSSSCGSGSDWLTQELDATGLQRMKWVENNYMVYNYCADVQRFPQGLPAECNATS

>SeXTH2

AFSTADFNKDFAITWGNGRAKILNNGDDLTLSLDKASGSGFQSKNEYLFGKIDMQLKLVPGNSAGTVTAYYLSSQGPTHDEIDFEFLGNVTGEPYTLHTNVFAQGKGSREKQFHLWFDPTKDFHTYSILWNPERIVFSVDGTPIREHKNMESKGIPFPKNQPMRIYSSLWEADDWATQGGRVKTDWTHAPFTASYRNFNADACVWASGSSSCGSGSDWLTQELDATGLQRMKWVENNYMVYNYCADVQRFPQGLPAECNATS

>SeXTH3

SSSSIIFITLFVTTFIAFSTADFNKDFAITWGDGRAKILNNGDDLTLSLDKASGSGFQSKNEYLFGKIDMQLKLVPGNSAGTVTAYYLSSQGPTHDEIDFEFLGNVTGEPYTLHTNVFAQGKGSREKQFHLWFDPTKNFHTYSILWNPERIVFSVDGTPIREHKNMESKGIPFPKNQPMRIYSSLWEADDWATQGGRVKTDWTHAPFTASYRNFNADACVWASGSSSCGSGSAPKSGSDWLTQELDATGLQRMKWVENNYMVYNYCADVQRFPQGLPAECNATS

>SeXTH4

ASMIAMASGVGDLNQNFDITWGDGRAKILNNGQLLTLSLDKTSGSGFQSKNEYLFGKIDMQIKLVKGNSAGTVTAYYLSSQGPTHDEIDFEFLGNVTGEPYTLHTNVFTQGKGGREQQFRLWFDPTVDFHTYSILWNPQSIVFYVDKTPIRVFKNMETQGIPFPKNQPMKIYSSLWDAEDWATQGGRVKTDWSKAPFMASYRNFNAEDSCSVNSSNGRSTCT

SASSWMTQQLDTTSQSRLKWAQKNYMIYNYCTDAPRFPQGFPKECSV

>SeXTH5

LMASSIFYILLIASMIAMASGVGDLNQNFDITWGDGRAKILNNGQLLTLSLDKTSGSGFQSKNEYLFGKIDMQIKLVKGNSAGTVTAYYLSSQGPTHDEIDFEFLGNVTGEPYTLHTNVFTQGKGGREQQFRLWFDPTVDFHTYSILWNPQSIVFYVDKTPIRVFKNMETQGIPFPKNQPMKIYSSLWDAEDWATQGGRVKTDWSKAPFMASYRNFNAEDSCSVNSSNGRSTCTSASSWMTQQLDTTSQSRLKWAQKNYMIYNYCTDAPRFPQGFPKECSV

>SeXTH6

ILNNGRLLTLSLDKTSGSGFQSKNEYLFGKIDMQIKLVKGNSAGTVTAYYLSSQGPTHDEIDFEFLGNVTGEPYTLHTNVFTQGKGGREQQFRLWFDPTVDFHTYSILWNPQSIVFYVDKTPIRVFKNMETQGIPFPKNQPMKIYSSLWDAEDWATQGGRVKTDWSKAPFMASYRNFNAEDSCSVNSSNGRSTCTSASSWMTQQLDTTSQSRLKWAQKNYMIYNYCTDAPRFPQGFPKECSV

>SeXTH7

LMAPSIFYILLIASMIAMAFGAGDLNQNFDITWGDGRAKILNNGQLLTLSLDKTSGSGFQSKNEYLFGKIDMQIKLVKGNSAGTVTAYYLSSQGPTHDEIDFEFLGNVTGEPYTLHTNVFTQGKGGREQQFRLWFDPTTDFHTYSILWNPQSIVFYVDKTPIRVFKNMETQGISFPKNQPMKIYSSLWDAEDWATQGGRVKTDWSKAPFMASYRNFNAEDSCSVNSSNGRSTCTSASSWMTQQLDTTSQSRLKWAQKNYMIYNYCTDAPRFPQGFPKECSVPQ

>SeXTH8

LMAPSIFYILLIASMIAMAFGAGDLNQNFDITWGDGRAKILNNGQLLTLSLDKTSGSGFQSKNEYLFGKIDMQIKLVKGNSAGTVTAYYLSSQGPTHDEIDFEFLGNVTGEPYTLHTNVFTQGKGGREQQFRLWFDPTTDFHTYSILWNPQSIVFYVDKTPIRVFKNMETQGISFPKNQPMKIYSSLWDAEDWATQGGRVKTDWSKAPFMASYRNFNAEDSCSVNSSNGRSTCTSASSWMTQQLDTTSQSRLKWAQKNYMIYNYCTDAPRFPQGFPKECSVPQ

>SeXTH9

FYVDKTPIRVFKNMETQGISFPKNQPMKIYSSLWDAEDWATQGGRVKTDWSKAPFMASYRNFNAEDSCSVNSSNGRSTCTSASSWMTQQLDTTSQSRLKWAQKNYMIYNYCTDAPRFPQGFPKECSV

>SeXTH10

TMKILKNMIVSFFFFALVFSVTISAKPAKFQQDFQVTWAENHLKHVDGDRVVQLKLDQNSGCGFASKYRYHFGSVSMKIKLVPGDSAGTVTAFYMNSDTDTIRDELDFEFLGNRTGQPYTVQTNIFTNGTGEREQRVNLWFDPSTDFHTYTILWNHNHTVFYVDEVPIRVYKNHEAIGVPYPKAQPMGVFSTLWEADDWATCGGKEKIDWSKAPFYAYYKDFEIEGCTSPGPLTCASDQQNWWESATYKELTPTQAKLYRWVRNNHMIYDYCNDISRFLVTPPEC

>SeXTH11

TMKILKNMIVSFFFFALVFSVTISAKPAKFQQDFQVTWAENHLKHVDGDRVVQLKLDQNSGCGFASKYRYHFGSVSMKIKLVPGDSAGTVTAFYMNSDTDTIRDELDFEFLGNRTGQPYTVQTNIFTNGTGEREQRVNLWFDPSTDFHTYTILWNHNHTVFYVDEVPIRVYKNHEAIGVPYPKAQPMGVFSTLWEADDWATCGGKEKIDWSKAPFYAYYKDFEIEGYTSPGPLTCASDQQNWWESATYKELTPTQAKLYRWVRNNHMIYDYCNDISRFLVTPPEC

>SeXTH12

ISLLNFLTVVSNAAFNLPTTTFDDGYSPLFADFNIKRSEDGRSANLHLNRYAGSGFISSKYYDHGFFSSRIKLPGNYTAGVVVAFYTSNGDVWEKHHDELDIEFLGNLKGKPWRFQTNMYGNGSTSRGREERYRLWFDPSKDFHQYSILWTPKHIIFYVDNVPIREIIRNEAMGSDYPSKPMSLYATIWDASSWATSGGRHKVKYEFEPFVSEFTEFMLDGCPVDPIEQVAGTTGSLDCTTKKNEIENKEYSEITPRGQKAMKWFREKYMYYSYCYDTVRYSVPPPECVIVPSEKERFKETGRLKFGSIPRRQRKRRPRGHGRKPNNNVESKGLKANM

>SeXTH13

NILLYISLLNFLTVVSNAAFNLPTTTFDDGYSPLFADFNIKRSEDGRSANLHLNRYAGSGFISSKYYDHGFFSSRIKLPGNYTAGVVVAFYTSNGDVWEKHHDELDIEFLGNLKGKPWRFQTNMYGNGSTSRGREERYRLWFDPSKDFHQYSILWTPKHIIFYVDNVPIREIVRNEAMGSDYPSKPMSLYATIWDASSWATSGGRHKVKYEFEPFVSEFTEFVLDGCPVDPIEQVAGTTGSLDRTTKKSEIENKEYSEITPRGQKAMKWFREKYMYYSYCYDTVRYSVPPPECVIVPSEKERFKETGRLKFGSIPRRQRKRRPRGHGRKPNNNVESKGLKANM

>SeXTH14

LSSEGKTHDEIDFEFLGNVTGQPYTLHTNVFTQGEGGREQQFHLWFDPTKKFHTYSVIWSHRMIIFLVDNTPIRVFKNHEARGVAFPKKQPMKIYSSLWNADQWATQGGRVKTDWSKAPFTAYYRRFNVNPNHVAAVAAYTDDKLQNQQQLNAWSRRRLRWVQKYYMIYNYCTDAQRFPRGIPRECKMI

>SeXTH15

MGVSSNGLLVIMVMLVALALSTTSVNGGNFDQDFEVTFGDGRVKKFWRGQMLTLSLDSVSGSGFKSKRSYLFGRIDMQLKLVAGNSAGTVTAYYLSSEGKTHDEIDFEFLGNVTGQPYTLHTNVFTQGEGGREQQFHLWFDPTKKFHTYSVIWSHRMIIFLVDNTPIRVFKNHEARGVAFPKKQPMKIYSSLWNADQWATQGGRVKTDWSKAPFTAYYRRFNVNPNHVAAVAAYTDDKLQNQQQLNAWSRRRLRWVQKYYMIYNYCTDAQRFPRGIPRECKMI

>SeXTH16

IYMNRSSSSSCSLCSSHNFIVLAVILCVYFTFQSSITLHEKTLPSDEQQIKTSSFLSSRKFDFNNDIRSSVDQPPPHHFSYQTSTIIDQPSLYAHDVKEETSSSSFQPPSQIGNFIEPQITQGLAKDFDITWGDHRGKFYENGDLLSLSLDKYSGSGFQSKNEYLFAKIDMKIKLISDNSAGTVTTFYMSSNGTSHDEIDFEFLGNVSGQPYTIHTNVYCQGRGNREKQFYLWFDPTTDFHTYSILWNPKRIIFFVDGTPIRVFENMEPFGVPFPNRQPMRIYSSLWNADDWATQGGRVHTDWNQAPFVASFRDFSTSSCVWSYGSSTCGLDPRPSGFENAWLGEELDASGLEKMRWVQDNYMTYNYCKDTWRFHDGLPKECTI

>SeXTH17

IYMNRSSSSSCSLCSSHNFIVLAVILCVYFTFQSSITLHEKTLPSDEQQIKTSSFLSSRKFDFNNDIRSSVDQPPPHHFSYQTSTIIDQPSLYAHDVKEETSSSSFQPPSQIGNFIEPQITQGLAKDFDITWGDHRGKFYENGDLLSLSLDKYSGSGFQSKNEYLFAKIDMKIKLISDNSAGTVTTFYMSSNGTSHDEIDFEFLGNVSGQPYTIHTNVYCQGRGNREKQFYLWFDPTTDFHTYSILWNPKRIIFFVDGTPIRVFENMEPFGVPFPNRQPMRIYSSLWNADDWATQGGRVHTDWNQAPFVASFRDFSTSSCVWSYGSSTCGLDPRPSGFENAWLGEELDASGLEKMRWVQDNYMTYNYCKDTWRFHDGLPKECTI

>SeXTH18

MKVGIFCLWFGIGLMLEVVSSSKFDELFKPTWALDHFIYEGESLKLKLDNFSGAGFQSKSKYLYGKVTVQIKLVEGDSAGTVTAYYMSSEGPTHNEFDFEFLGNTTGEPYVVQTNIYVNGEGNREQRLNLWFDPTKDFHNYSLLWTPRKVVFMVDETPIREHTNLEHKGIPYPKDQAMGVYSSIWNADDWATQGGRVKTDWSHAPFIASYKGFEIDACECPSVPTTATADIMKRCSSYSNNTPTTSSSTSSELTVHQSHQLMWVKANHLIYDYCTDTTRFPTIPAECVHHRH

>SeXTH19

MKVGIFCLWFGIGLMLEVVSSSKFDELFKPTWALDHFIYEGESLKLKLDNFSGAGFQSKSKYLYGKVTVQIKLVEGDSAGTVTAYYMSSEGPTHNEFDFEFLGNTTGEPYVVQTNIYVNGEGNREQRLNLWFDPTKDFHNYSLLWTPRKVVFMVDETSIREHTNLEHKGIPYPKDQAMGVYSSIWNADDWATQGGRVKTDWSHAPFIASYKGFEIHACECPGVPTTATADIMKRCSSYSNSTSTSTTSSELTIHQSHQLMWVKANHLIYDYCTDTTRFPTIPAECVHHRH

>SeXTH20

IAFSTADFNKDFAITWGDGRAKILNNGDDLTLSLDKASGSGFQSKNEYLFGKIDMQLKLVPGNSAGTVTAYYLSSQGPTHDEIDFEFLGNVTGEPYTLHTNVFAQGKGSREKQFHLWFDPTKNFHTYSILWNPERIVFSVDGTPIREHKNMESKGIPFPKNQPMRIYSSLWEADDWATQGGRVKTDWTHAPFTASYRNFNADACVWASGSSSCGSGSAPKSGSDWLTQELDATGLQRMKWVENNYMVYNYCADVQRFPQGLPAECTTKA

>SeXTH21

IAFSTADFNKDFAITWGDGRAKILNNGDDLTLSLDKASGSGFQSKNEYLFGKIDMQLKLVPGNSAGTVTAYYLSSQGPTHDEIDFEFLGNVTGEPYTLHTNVFAQGKGSREKQFHLWFDPTKNFHTYSILWNPERIVFSVDGTPIREHKNMESKGIPFPKNQPMRIYSSLWEADDWATQGGRVKTDWTHAPFTASYRNFNADACVWASGSSSCGSGSAPKSGSDWLTQELDATGLQRMKWVENNYMVYNYCADVQRFPQGLPAECTTKA

>SeXTH22

MGCSRQRCIIKLVLFFCVYKEIIAHSESINKLPIISFDEGYSHLFGDNNLMVVRDGKSVLLSLDERTGSGFMSHDLYLHGFFSASMKLPSDYTAGVVVAFYMSNADMYEHNHDELDIEFLGNIRGKEWRVQTNVYGNGSTSVGREERYTLWFDPSEEFHRYSILWAEDKIIFYVDDVPIREIKRTEAMKKDFPSKPMSLYTTIWDASTWATNGGKYKVDYRYAPYVAEFSDLVLHGCSVDPIERSGRCDDVGSIDGSIPSGITPSQRTEMANYRKKFLTYSYCYDQSRYKTPPSECVIDPREADRLKRFDPVTFGGSRRHRGSHHRHHHNHRGSTKNEKASI

>SeXTH23

ILSNNKYDNVMLSIVMIVALMLSSKAYGAGNFNNEFEPTFGGERVRVSGRGGQELTLSLDQGSGSGFRSKKDYLFGRVDMQMKFVGGNSAGTVTTLYLSSDQSTGRHDEIDFEFLGNVSGQPYTIHTNVFSQGEGSREQQFHLWFDPTRNFHTYSVVWNPKLIMFLVDETPLRIFRNYEDRGISFFPKNQRQKIYASLWEADDWATQGGAVKTDWSKAPFTAYYRNFNIDNSPNGRYMHHDLDANSRRRLRWVQKNYMIYNYCSDWKRFSQAFPPECKHSKV

>SeXTH24

MGILSNNKYDNVMLSIVMIVALMLSSKAYGAGNFNNEFEPTFGGERVRVSGRGGQELTLSLDQGSGSGFRSKKDYLFGRVDMQMKFVGGNSAGTVTTLYLSSDQSTGRHDEIDFEFLGNVSGQPYTIHTNVFSQGEGSREQQFHLWFDPTRNFHTYSVVWNPKLIMFLVDETPLRIFRNYEDRGISFFPKNQRQKIYASLWEADDWATQGGAVKTDWSKAPFKAYYRNFNIDNSPNGRYMHHDLDANSRRRLRWVQKNYMIYNYCSDWKRFSQAFPPECKHSKV

>SeXTH25

IMLLGVTFMIIVPMTISATSWPPSPGYYPSKKFRSMRFSKGFKNLWGPQHQRLNNHALSLWLDRSSGSGFKSVRPFRSGYFGTSIKLQQGYTAGVITSFYLSNNEAHPGHHDEIDMEFLGTTFGKPYVLQTNVYITGSGDGNIIGREMKFHLWFDPTQGFHHYAIFWSPNEIIFLVDDIPIRRYARKSAATFPRRPLWLYGSIWDASDWATENGKYKANYNYQPFVGQYTNFKASGCSAYAPLSCRPVSVSPSRSGGLSRKQLYVMKWVQKYYKIYDYCKDRKKNHALTPECWLRG

>SeXTH26

IAFSTADFNKDFAITWGDGRAKILNNGDDLTLSLDKASGSGFQSKNEYLFGKIDMQLKLVPGNSAGTVTAYYLSSQGPTHDEIDFEFLGNVTGEPYTLHTNVFAQGKGSREKQFHLWFDPTKDFHTYSIVWNPERIVFSVDGTPSGNTRIWNQRESHSL

>SeXTH27

IAFSTADFNKDFAITWGDGRAKILNNGDDLTLSLDKASGSGFQSKNEYLFGKIDMQLKLVPGNSAGTVTAYYLSSQGPTHDEIDFEFLGNVTGEPYTLHTNVFAQGKGSREKQFHLWFDPTKDFHTYSIVWNPERIVFSVDGTPSGNTRIWNQRESHSL

>SeXTH28

KGTLTVWLDRSSGSGFKSLQRFRSGYFGSAMKLQPGYTAGVITSFYLSNNEDFPGHHDEVDIEFLGTTPGKPYVLQTNVYIRGSGDGTLIGREMKFNLWFDPTKGFHHYAIFWSPSDIIFFVDDVPIRRYPRKSDATFPVRPMSLYGSIWDASSWATEDGKYKANYNYQPFVGQYRDFKIVACSSGGPCSRPKVSPLRSNMLSPQQLSAMKWAQSTHLVYDYCRDSSRDHTLTPEC

>SeXTH29

MATFLCVIIAFIFMSNLSNAQLSPGYYPSNKIASLSFNQGFRTLWGPQHQRFQQGTLTVWLDRSSGSGFKSLQRFQSGYFGAAMKLQPGYTAGVITSFYLSNNEDFPGHHDEVDIEFLGTTPGKPYVLQTNVYVRGSGDGTLIGREMKFNLWFDPTKGFHHYAIFWSPSHIIFFVDDVPIRRYPRKSDATFPVRPMSLYGSIWDASSWATEDGKYKANYNYQPFVGQYRDFKIVACTSGGPCSRPKVSPLRSNLLSPQQLSAMKWAQSTHLVYDYCRDSSRDHTLTPEC

>SeXTH30

FGKIDMQLKLVPGNSAGTVTAYYLSSQGSTHDEIDFEFLGNVTGEPYTLHTNVFAQGKGSREKQFHLWFDPTKDFHTYSILWNPERIVFSVDGTPIREHKNMESKGIPFPKNQPMRIYSSLWEADDWATQGGRVKTDWTHAPFTASYRNFNADACIWASGSSSCGSGSAPKSGSDWLTQELDATGLQRMKWVENNYMVYNYCADVQRFPEGLPTEC

>SeXTH31

GRAKILNNGDDLTLSLDKASGSGFQSKSEYLFGKIDMQLKLVPGNSAGTVTAYYLSSQGPTHDEIDFEFLGNVTGEPYTLHTNVFAQGKGSREKQFHLWFDPTKDFHTYSILWNPERIVFSVDGTPIREHKNMESKGIPFPKNQPMRIYSSLWEADDWATQGGRVKTDWTHAPFTASYRNFNADACVWASGSSSCGSGSAPKSGSDWLTQELDATGLQRMKWVENNYMVYNYCADVQRFPEGLPAEC

>SeXTH32

VMLQVSLTHSTRTSLLRAKAVVKNSFTYGSTPLKISTPAILWNPERIVFSVDGTPIREHKNMESKGIPFPKNQPMRIYSSLWEADDWATQGGRVKTDWTHAPFTASYRNFNADACVWASGSSSCGSGSAPKSGSDWLTQELDATGLQRMKWVENNYMVYNYCADVQRFPEGLPAEC

>SeXTH33

IAFSTADFNKDFAITWGDGRAKILNSGDDLTLSLDKASGSGFQSKNEYLFGKIDMQLKLVPGNSAGTVTAYYLSSQGPTHDEIDFEFLGNVTGEPYTLHTNVFAQGKGSREKQFHLWFDPTKDFHTYSIVWNPERIVFSVDGTP

>SeXTH34

IAFSTADFNKDFAITWGDGRAKILNSGDDLTLSLDKASGSGFQSKNEYLFGKIDMQLKLVPGNSAGTVTAYYLSSQGPTHDEIDFEFLGNVTGEPYTLHTNVFAQGKGSREKQFHLWFDPTKDFHTYSIVWNPERIVFSVDGTP

>SeXTH35

DFAITWGDGRAKILNSGDDLTLSLDKASGSGFQSKNEYLFGKIDMQLKLVPGNSAGTVTAYYLSSQGPTHDEIDFEFLGNVTGEPYTLHTNVFAQGKGSREKQFHLWFDPTKDFHTYSIVWNPERIVFSVDGTPSGNTRIWNQRESHSL
